# The man, the plant, and the insect: shooting host specificity determinants in *Serratia marcescens* pangenome

**DOI:** 10.3389/fmicb.2023.1211999

**Published:** 2023-09-12

**Authors:** Anton E. Shikov, Anastasiya V. Merkushova, Iuliia A. Savina, Anton A. Nizhnikov, Kirill S. Antonets

**Affiliations:** ^1^Laboratory for Proteomics of Supra-Organismal Systems, All-Russia Research Institute for Agricultural Microbiology, St. Petersburg, Russia; ^2^Faculty of Biology, St. Petersburg State University, St. Petersburg, Russia

**Keywords:** *Serratia marcescens*, pangenome, host specificity, virulence, pan-GWAS, human infections, PGP

## Abstract

**Introduction:**

*Serratia marcescens* is most commonly known as an opportunistic pathogen causing nosocomial infections. It, however, was shown to infect a wide range of hosts apart from vertebrates such as insects or plants as well, being either pathogenic or growth-promoting for the latter. Despite being extensively studied in terms of virulence mechanisms during human infections, there has been little evidence of which factors determine *S. marcescens* host specificity. On that account, we analyzed *S. marcescens* pangenome to reveal possible specificity factors.

**Methods:**

We selected 73 high-quality genome assemblies of complete level and reconstructed the respective pangenome and reference phylogeny based on core genes alignment. To find an optimal pipeline, we tested current pangenomic tools and obtained several phylogenetic inferences. The pangenome was rich in its accessory component and was considered open according to the Heaps’ law. We then applied the pangenome-wide associating method (pan-GWAS) and predicted positively associated gene clusters attributed to three host groups, namely, humans, insects, and plants.

**Results:**

According to the results, significant factors relating to human infections included transcriptional regulators, lipoproteins, ABC transporters, and membrane proteins. Host preference toward insects, in its turn, was associated with diverse enzymes, such as hydrolases, isochorismatase, and N-acetyltransferase with the latter possibly exerting a neurotoxic effect. Finally, plant infection may be conducted through type VI secretion systems and modulation of plant cell wall synthesis. Interestingly, factors associated with plants also included putative growth-promoting proteins like enzymes performing xenobiotic degradation and releasing ammonium irons. We also identified overrepresented functional annotations within the sets of specificity factors and found that their functional characteristics fell into separate clusters, thus, implying that host adaptation is represented by diverse functional pathways. Finally, we found that mobile genetic elements bore specificity determinants. In particular, prophages were mainly associated with factors related to humans, while genetic islands-with insects and plants, respectively.

**Discussion:**

In summary, functional enrichments coupled with pangenomic inferences allowed us to hypothesize that the respective host preference is carried out through distinct molecular mechanisms of virulence. To the best of our knowledge, the presented research is the first to identify specific genomic features of *S. marcescens* assemblies isolated from different hosts at the pangenomic level.

## 1. Introduction

A rod-shaped Gram-negative bacterium, *Serratia marcescens*, belonging to the *Enterobacteriaceae* family, is ubiquitously found in various environmental niches, including soil, freshwater, and air (Matteoli et al., 2021) and often isolated from animals and plants as well (Mahlen, 2011). The description of the species dates back to 1,819 in Italy as red spots on polenta (Merlino, 1924). Since this bacterium was initially considered non-pathogenic and produced red pigment, it found wide application as a biological warfare test organism, which led to the release of the bacterium by the US government from the 1940s to the 1960s with a view of monitoring possible bioterrorism threats (Mahlen, 2011). Later on, however, it has become clear that *S. marcescens* is a causative agent of nosocomial infections, especially in neonates and immunocompromised patients (Moradigaravand et al., 2016; Saralegui et al., 2020). Clinical studies illustrated that *S. marcescens* is capable of invading the respiratory (Dessì et al., 2009) and urinary tracts (Lancaster, 1962), wounds (Moradigaravand et al., 2016), and bloodstream (Haddy et al., 1996). It is worth mentioning that bacteremia, in the case of bloodstream invasion, is a severe disease with a mortality rate exceeding 20% (Haddy et al., 1996). The half-century history of epidemiological monitoring brought evidence of multiple infection outbreaks caused by the bacterium (Escribano et al., 2019; Saralegui et al., 2020). Due to the emergence of antibiotic-resistant strains driven by HGT (horizontal gene transfer), studying genomic features of pathogenic *S. marcescens* isolates is of great importance for the healthcare system (Matteoli et al., 2021; Shikov et al., 2022). Several *S. marcescens* strains are pathogenic to bovines (Friman et al., 2019), birds (Saidenberg et al., 2007), reptiles (Pye et al., 1999), and fishes (Baya et al., 1992).

The repertoire of hosts affected by *S. marcescens* is not limited to vertebrates. Being present in the insect gut, it could be efficiently transmitted between individuals (Dupriez et al., 2022). Virulence potential is delineated by host species, development stage, and environmental conditions (Dupriez et al., 2022). The severity of the disease also depends on the abundance of *S. marcescens* in the host’s microbiome (Sikorowski et al., 2001). Apart from insecticidal activity, *S. marcescens* exerts an effect on plants which can be either pathogenic or stimulatory. Recent studies have provided multiple evidence that *S. marcescens* is an opportunistic plant pathogen inducing black rot of sweet orange (*Citrus sinensis* L.) (Hasan et al., 2020), cucurbit yellow vine disease (CYVD) (Besler and Little, 2017), necrotic lesions on oleander (*Nerium oleander* L.) leaves (Fodor et al., 2022), bell pepper (*Capsicum annuum* L.) soft-rot (Gillis et al., 2014). On the other hand, some *S. marcescens* strains exhibit plant growth-promoting (PGP) properties (Matteoli et al., 2018; Abreo and Altier, 2019). These features include induction of phosphorus solubilization (Tripura et al., 2007), defense from insects by chitinases (Vaikuntapu et al., 2016) coupled with prodigiosin biocontrol (Suryawanshi et al., 2015), and antagonism with fungal plant pathogens (Li et al., 2015; Matteoli et al., 2018).

Studying the pathogenicity of *S. marcescens* as a model object has expanded our understanding of the infection process mediated by various virulence factors. These included secreted proteases (Kamata et al., 1985), extracellular phospholipases (Shimuta et al., 2009), hemolysins (ShlA) (Abreo and Altier, 2019), iron acquisition transporters (Létoffé et al., 1994), and lipopolysaccharides (Kurz et al., 2003). At the same time, the mechanisms affecting specificity and adaptation to certain hosts are poorly studied. General functional peculiarities of putative specificity factors remain unknown as well. Approaches that seem promising to reveal these determinants are comparative genetics and pangenomics. With the advent of high-throughput sequencing technologies, it has become possible to analyze bacteria on a whole-genome scale. In 2013, the first complete genome of *S. marcescens* WW4 was sequenced (Chung et al., 2013), and since then, publically available databases are continually updating with new complete genome assemblies. The usage of genomic datasets made it possible to examine particular groups of genomes within species’ pangenomes. For example, it allowed characterizing the spread of hospital isolates of *S. marcescens* across the United Kingdom and Ireland (Moradigaravand et al., 2016), identifying antibiotic-resistance factors within historical lineages (Saralegui et al., 2020; Matteoli et al., 2021), and comparing *S. marcescens* with other obligate symbiotic species within the genus (Li et al., 2015; Chen et al., 2017). A recent large-scale pangenomic study enabled defining the population structure of the *Serratia* genomes corresponding to phenotypical traits and ecological niches in which strains resided and showed the relationships between the genetic flow and the emergence of new isolates (Williams et al., 2022).

Despite large progress made over the last few years, pangenomics approaches were not applied to identify putative factors delineating hosts’ preference as more attention was paid to virulence rather than specificity. On that account, we reconstructed the *S*. *marcescens* pangenome using 73 genome assemblies of complete level. In doing so, we compared several popular tools and applied different phylogenetic approaches to develop the most optimal pipeline. Furthermore, we predicted sets of putative specificity factors associated with strains isolated from humans, insects, and plants and carried out functional annotation to characterize functional features of the respective determinants.

## 2. Materials and methods

### 2.1. Data acquisition

The genome assemblies of *S. marcescens* were downloaded from the NCBI Assembly database (Kitts et al., 2016). Only the genomes deposited in the RefSeq database (O’Leary et al., 2016) with the “complete genome” assembly level remained for further analysis. In order to obtain a high-quality dataset for pangenome analysis, we discarded assemblies with excessively high similarity. We used Assembly-Dereplicator^1^ with 99% of identity and a sketch size of 100,000 and selected reference assemblies from clusters accordingly. The respective metadata of the assemblies, including the number of contigs and CDS (coding DNA sequences), genome size, and N50, were obtained using the “ESummary” command from the Entrez v.7.40.20170928+ds-1 package. Next, we calculated the mean GC content of the genomes using a Python v.3.6.9 script implementing the Biopython v1.73 library (Cock et al., 2009). To assess the completeness of the taxonomic markers in the genomes, the BUSCO v5.4.3 package (Simão et al., 2015) was applied with the “*Enterobacteriales”* order specified.

### 2.2. Pangenome reconstruction

Three popular tools for pangenome were applied, namely, Roary (Page et al., 2015), Panaroo (Tonkin-Hill et al., 2020), and PEPPAN (Zhou et al., 2020). All the programs were launched with default settings and a 95% identity threshold for defining core genes. To check whether the pangenome is open or closed, we transformed gene presence/absence tables into binary matrices and applied the micropan package for the R v.3.6.3 programming language (Snipen and Liland, 2015) to calculate the alpha value based on the Heaps’ law. Moreover, we plotted the power-fit curves with a custom R script implementing the ggplot2 library (Wickham, 2016) with 1,000 permutations to calculate the number of genes after expanding the pangenome with new assemblies.

### 2.3. Phylogenetic analysis

We tested three whole genome-based phylogeny reconstruction approaches to choose the optimal pipeline. First, we picked the trees based on the patterns of the presence/absence of accessory genes. Roary and PEPPAN provided the respective trees as the output. To build the corresponding tree from the Panaroo-reconstructed pangenome, we transformed the gene presence/absence table to pseudoalignment with present genes marked as “C” and absent genes marked as “A” with a custom Python script. The phylogenetic tree was obtained using the FastTree v.2 program with 1,000 bootstrap replications (Price et al., 2010). Second, we obtained core gene alignments. Two aligners were applied when reconstructing pangenomes, namely MAFFT (Katoh and Standley, 2013) and Prank (Löytynoja, 2014). Core SNPs (single nucleotide polymorphisms) were selected with the SNP-sites v2.5.1 tool (Page et al., 2016). We used two types of alignments, namely, concatenated alignments with a single partition specifying the optimal evolutionary model and alignments with the respective individual partitions for each gene cluster. Best-fit evolutionary models were selected using the ModelTest-NG v0.1.7 (Darriba et al., 2020) in the “ml” mode with the best BIC value (Bayesian information criterion). ML (maximum likelihood) phylogenetic trees were built with the RAxML-NG v1.1.0 program (Kozlov et al., 2019) with 1,000 bootstrap replications. Third, we clusterized genomes based on the ANI (average nucleotide identity). Matrix with the pair-wise ANI values was obtained using the Mash v2.3 utility (Ondov et al., 2016) with a k-mer size of 21 and a sketch size of 100,000. The genome clusterization was performed with the “Bclust” function from the shipunov package for R^2^ with Euclidean distance metrics and the “complete” clustering method. We then characterized the similarity between the reconstructed trees and assessed the quality of these trees. All the reconstructed trees were topologically compared via the tqDist v1.0.2 library with quartet distance metrics (Sand et al., 2014). Calculated quartet distances were presented as a matrix that was visualized with the ggplot2 package. The quality of the trees was characterized based on two features, namely, mean support of the nodes, and tree balance indices. Three balance indices such as “CollesLike,” “Sackin,” and “Cophenetic” were calculated using the v2.0 package for R (Mir et al., 2018). The measurements were then plotted on the plane with mean node support on the x-axis and the sum of balance indices divided by the maximum value for each index on the y-axis.

### 2.4. Virulence and specificity factors analysis

To identify commonly known virulence factors in the analyzed genomes, we used the MMseqs2 tool (Steinegger and Söding, 2017) against the database with protein sequences of virulence factors from VFDB (virulence factor database) (Chen et al., 2005). For each query, the best hit from the database according to the e-value was selected. Next, we retained the hits with not less than 70% query and target coverage coupled with a 70% identity threshold. We then downloaded the metadata of the assemblies from the NCBI BioSample database (Barrett et al., 2012). The genomes were classified according to the infected host, namely, 37 strains were isolated from humans, 4–insects, and 3–plants as well as 29 unassigned strains. Next, binary tables of traits (affected host) were generated using a custom Python script. The data was used to reveal candidate genes associated with host specificity with Scoary v1.6.16 (Brynildsrud et al., 2016). Next, we selected positive associations by calculating the percentage of positive and negative hits using the following formula (1) and then picked only those associations for which the positive ratio was higher than the negative ratio:


(1) $$\frac{p\,p}{\left(p\,p+p\,n\right)}>\frac{n\,p}{\left(n\,n+n\,p\right)}$$

where *pp* and *pn* – stand for the number of present and absent genes in genomes attributed to a particular host, respectively, while *np* and *nn* represent the respective numbers for genomes attributed to other hosts or lacking annotations accordingly.

Scoary-predicted sets of genes were then matched with found virulence factors to identify possible novel virulence determinants. We utilized the gene presence/absence table from the Panaroo-based pangenome and the best ML phylogeny to detect patterns of gene co-occurrence with Coinfinder v1.2.0 (Whelan et al., 2020). Next, tentative host specificity factors were matched with the connected components from the co-occurrence graph.

### 2.5. Functional annotation

Functional annotation of the protein sequences was carried out with eggNOG standalone tool v2.0.1b-2-g816e190 in the “mmseqs2” search mode (Huerta-Cepas et al., 2017). The attributions belonging to the COG (Cluster of Orthologous Genes) (Tatusov et al., 2000) and GO (Gene Ontology) (Ashburner et al., 2000) annotation systems were examined with the latter including three classes, namely, cellular component (CC), molecular function (MF), and biological process (BP). The annotations of KEGG (Kyoto Encyclopedia of Genes and Genomes) terms were downloaded from the KEGG (Kanehisa and Goto, 2000) site.^3^ The text representation was converted to a CSV table using a custom Python script. To characterize functional features of the gene groups (core/accessory, virulence, and host specificity determinants), we applied the custom Python script implementing a hypergeometric test within COG and KEGG annotation systems considering KO codes for the latter. We first build universes for each annotation system with the number of occurrences for each term. Next, we calculated the *p*-values of enrichments related to a certain term for each gene group using the hypergeometric test. The lists of *p*-values were corrected with FDR adjustment (false discovery rate). GO enrichments were calculated utilizing the topGO v.3.15 package (Alexa and Rahnenfuhrer, 2022). We then chose significant enrichments according to adjusted q-values. After that, we performed k-means clustering of significant enrichments. We first obtained a distance matrix based on the pair-wise Szymkiewicz–Simpson (the number of intersections divided by the minimum length of the respective set) and Jaccard coefficients (the number of intersections divided by the length of the union). The optimal number of clusters for the k-means procedure was evaluated with the elbow method (Demidenko, 2018) by manual inspection of depicted with-in-Sum-of-Squares (WSS) numbers. Clustering patterns were subsequently visualized via the “autoplot” function from ggfortify v0.4.11 (Tang et al., 2016).

### 2.6. Mobile genomic elements search

We described the genomic landscape of *S. marcescens* by identifying mobile genetic elements in the analyzed genomes. Insertions sequences were found using the ISEScan v1.7.2.3 software (Xie and Tang, 2017). To detect prophages, Phigaro v2.3.0 was applied (Starikova et al., 2020). Finally, we revealed genomic islands (GIs) with IslandPath-DIMOB utility (Bertelli and Brinkman, 2018). The CSV tables made were then transformed into coordinates of the respective genetic elements in BED format. Similarly, BED files corresponding to the detected virulence and host-specificity factors were generated using a custom Python script. BED files pertaining to factors and genetic elements were then intersected using the BEDtools “intersect” utility (Quinlan and Hall, 2010) to reveal associations between mobile genetic elements and determinants of virulence and host specificity. We then collected the data on the abundance of different MGEs found in the assemblies attributed to a particular host and performed the ANOVA (analysis of variance) method followed by Tukey’s HSD (honestly significant difference) test to reveal statistical differences between genomes.

## 3. Results

### 3.1. Genomic properties of the dataset

**FIGURE 1:**
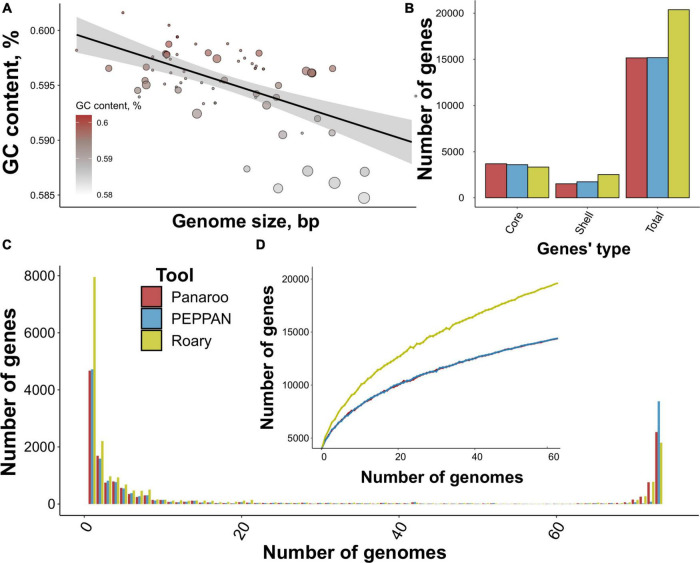
Properties of the analyzed dataset comprising *S. marcescens* genome assemblies and main characteristics of the reconstructed pangenome. **(A)** The relation between genome size and mean GC content. Dots’ size represents the number of contigs per assembly, and the intensity of the color codes GC content. A linear relationship between the properties is illustrated by a black line. **(B)** The number of gene groups (core, shell, and total) within pangenomes built using three tools, namely Panaroo, PEPPAN, and Roary. **(C)** U-curves of the respective pangenomes showing the frequency of pan-genes presented in a certain number of genomes. **(D)** The power-fit curve of *S. marcescens* pangenomes based on 1,000 permutations of a random sampling of genomes. Bars denote standard deviation.

We first downloaded 99 genome assemblies of *S. marcescens* from the NCBI assembly database (Kitts et al., 2016), and after the dereplication procedure, 73 assemblies remained. The percentage of present markers from the “*Enterobacteriales”* order exceeded 97.3% for all assemblies reaching 99.8% on average (Supplementary Table 1). The assemblies possessed similar genome length with the mean value of 5,297,794 b.p. harboring 4,843 CDS with 362 amino acid residues on average (Supplementary Figures 1A, B; Supplementary Table 2). Notably, the number of hypothetical proteins was proportional to the number of CDS (Supplementary Figure 1B) with approximately unannotated 313 proteins lacking per assembly (Supplementary Table 2). GC content of the genomes reached 59.5% on average (Supplementary Figure 1C; Supplementary Table 2). Interestingly, the GC content was inversely proportional to the genome size which also correlated with the number of contigs (Figure 1A). This could be explained by plasmids’ nucleotide compositions differing from chromosomes (Rocha and Danchin, 2002). The versatile GC content (from 58.5 to 60.2%) corroborated the reported phylogeny-dependent dispersion in GC and negative correlation with plasmids’ abundance as well (Ono et al., 2022). Other properties of analyzed genomes were commensurable indicating taxonomical relatedness and the validity of the dataset.

### 3.2. Assessing pipelines for pangenome analysis

To determine the most optimal pipeline for pangenome reconstruction, we first tested the most popular tools. Panaroo- and PEPPAN-based pangenomes exhibited very high similarity with 15,166 and 15,188 gene clusters (pan-genes) containing 3,700 and 3,600 core genes, while Roary reported a total of 20,380 pan-genes corresponding to 3,341 core genes, respectively (Figure 1B). The shape of U-curves reflecting the number of genes present in genomes was comparable for all pangenomes (Figure 1C; Supplementary Table 3). No considerable internal peaks indicated taxonomical homogeneity between the studied assemblies. Still, some remarkable differences were detected. First, the peak corresponding to gene clusters presented in all 73 genomes was the highest for PEPPAN-built pangenome (3,600 pan-genes vs. 2,523 for Panaroo). Second, Roary provided a considerably larger number of unique genes of 7,954 in comparison with Panaroo and PEPPAN reporting 4,674 and 4,720 genes, respectively.

All three power-fit curves did not reach saturation thus indicating that *S. marcescens* pangenome could be considered open. However, Roary provided a curve resembling linear function probably due to the inflation of unique genes (Figure 1D). We also calculated the α parameters according to Heaps’ law. The estimates spanned from 0.52 to 0.64 further proving the pangenome to be open as α < 1 implies openness (Tettelin et al., 2008).

In general, all the pangenomic approaches provided comparable results, however, PEPPAN and Panaroo provided a lesser number of accessory genes, thus minimizing their inflation which could lead to over-estimation of pangenome openness. However, PEPPAN’s limitation lies in the absence of pan-genes’ alignments making it impossible to build full-genome phylogeny. Therefore, we proposed Panaroo to be the most suitable tool for reconstructing the *S. marcescens* pangenome and, hence, considered the respective pangenome as the reference for further examination.

### 3.3. Core and accessory genes-based phylogenies

In addition to pangenome analysis, we also compared several phylogenomic pipelines. We considered three main approaches: using core gene alignment representing relationships between lineages (Ding et al., 2018), clustering accessory genes thus reflecting gene gain/loss and HGT (Whelan et al., 2021), and calculating average nucleotide identity (ANI) (Supplementary Figure 2) thought to reflect evolutionary history depending on overall genetic distance (Gosselin et al., 2022). Moreover, we either applied partition schemes for each core gene or utilized a single model.

We found that all the trees showed considerable topological similarity with a mean value of 92%. Importantly, phylogenies based on core genes were highly congruent with topological similarity exceeding 96% (Supplementary Figure 3A; Supplementary Table 4). On the other hand, clusterization-based trees were less consistent (Supplementary Figure 2A). Genome grouping according to ANI resembled core genome phylogenies more than those based on accessory genes’ distributions. In general, the highest mean similarity (94.5%) with all trees was shown for the phylogeny reconstructed on the basis of core genes obtained from the Panaroo-reconstructed pangenome aligned with MAFFT without partitions (Supplementary Figure 3B; Supplementary Table 4).

We also compared the quality of the reconstructed trees considering considered two measures, namely, mean branch support and tree balance. As it is evident from the graph, clusterizations gained higher support values than true core gene phylogenies and were more balanced as well (Supplementary Figure 3C; Supplementary Table 5). This observation, however, could be explained by less sophisticated computations applied to obtain such trees. The highest branch support was reported for the aforementioned phylogeny with the highest mean similarity, albeit being less balanced. Nevertheless, alignment-based phylogenies differed negligibly in terms of balance. According to our results, partitioning slightly increases tree balance but not branch support (Supplementary Figure 3C). Given that the former is not indicative of quality (Stam, 2002), we believe that an almost unnoticeable gain in balance does not cost such a considerable rise in computational cost (Supplementary Table 6). Taking into account all the facts mentioned, we selected a best-choice pipeline involving Panaroo for pangenome reconstruction and unpartitioned core gene alignment made by MAFFT for building a reference tree.

### 3.4. Distribution of host specificity and virulence factors

**FIGURE 2:**
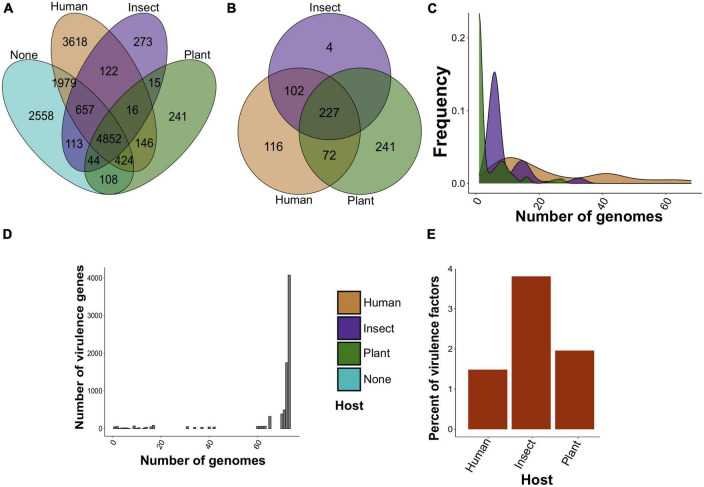
The number of found specificity and virulence factors in *S. marcescens* genome assemblies. **(A)** Venn diagram of common and non-shared gene clusters between assemblies attributed to a particular host (human, insect, and plant). Clusters are considered common if at least one gene from the orthologous cluster is shared by assemblies isolated from different hosts. “None” indicates the absence of the host in the metadata. **(B)** The same results for pangenome clusters reported as specificity factors using Scoary. **(C)** Frequency distribution of specificity factors. Plotted on the y-axis is the number of genomes in which the respective factor is present. **(D)** The abundance of virulence genes. The y-axis reflects the frequency of virulence factors possessed by a certain number of genomes. **(E)** Percentage of specificity factors having a respective homolog in the VFDB.

To reveal candidate specificity factors of *S. marcescens*, we attributed genomes to affected hosts specified in the BioSample database and found three main host groups, namely, humans, insects, and plants, with which 37, 4, and 3 assemblies were associated, respectively (Supplementary Table 7). The remaining 29 assemblies represented primarily environmental strains with unknown host specificity. We found that 4,852 core gene clusters were shared between all the groups (Figure 2A), whereas non-shared pan-genes reached 3,618, 241, and 273 for isolates infecting humans, plants, and insects, respectively. Interestingly, genomes related to human infection included more common gene clusters with those infesting plants than with insect-infecting (122 vs. 146), while the latter two groups shared 15 pan-genes only.

Given the unequal number of host-wise genomes, we selected only positive associations (see Section “Materials and methods”). Due to the scarcity of data, only 8 pan-genes related to human infection regarding Benjamini-Hochberg-corrected *p*-values we reported. These genes included surface lipoproteins and transporters (Supplementary Table 8). We, however, aimed to reveal general functional features of tentative specificity factors and thus selected all found hits based on raw *p*-values (Supplementary Table 9). There were only 3 reported gene clusters common for insect and plant infestations (Supplementary Figure 4A). When disclosing gene clusters and considering the existence of genes within pangenome clusters in host-attributed assemblies, we found that 227 gene clusters were common for all three groups, while 116, 4, and 241 pan-genes were unique for assemblies associated with humans, insects, and plants, respectively (Figure 2B). More specificity-related pan-genes were common for human- and insect-associated strains than for those infecting humans and plants (102 vs. 72), whereas groups attributed to plants and insects lacked shared clusters. After that, we analyzed the abundance of selected specificity markers among the genomes (Figure 2C). Plant-related gene clusters showed the lowest frequency with four genome assemblies on average compared with 9 and 24 genomes for putative specificity factors related to insect and human infections. In addition to this, plant-associated clusters contained 35% of hypothetical proteins in contrast to insect- and human-attributed clusters with 17 and 19%, respectively (Supplementary Figure 4B).

Next, we carried out the screening of known virulence factors from the VFDB (virulence factor database) among the analyzed genomes to find possible relations between virulence and specificity. We identified 7,955 virulence factors belonging to 231 pan-genomic clusters. On average, homologs of virulence factors were presented in 67 genomes, and the vast majority of factors represented core genes with a total of 6,714 (Figure 2D). The homologs showed 81% mean identity with the respective references from the database. Interestingly, three main groups of virulence genes in the context of the identity were found (Supplementary Figure 4C). They included core and shell genes with a mean identity of 80%, higher than 90%, or not exceeding 80%. Remarkably, a small fraction of virulence factors corresponded to specificity determinants. Only 81, 8, and 9 virulence genes constituted 1.5, 3.8, and 1.9% of specificity factors associated with humans, insects, and plants, respectively (Figure 2E).

**FIGURE 3:**
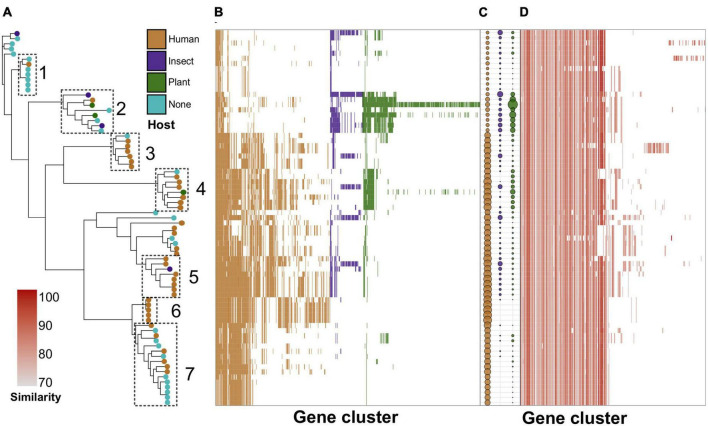
Phylogeny-wise distribution of specificity determinants and known virulence factors in *S. marcescens* genomes. **(A)** Reference ML (maximum likelihood) tree with leaves colored by the host. Dotted rectangles denote selected subclades with corresponding numbers assigned to them. **(B)** The presence/absence of specificity factors among the genomes in the heatmap ordered by the adjacent reference tree. **(C)** The number of host-wise specificity factors in a particular genome. The size of the dots is proportional to the number of respective genes. **(D)** Distribution of virulence factors in the genomic dataset. The intensity of the color denotes sequence similarity with the closest homolog from the VFDB (virulence factor database).

We then analyzed the phylogeny-wise distribution of these specificity factors (Figures 3A, B; Supplementary Table 10), and selected seven compact phylogenetic subclades forming distinct patterns in terms of specificity factors’ abundance (Figure 3A). These clades are also visible, albeit not explicitly when examining the distribution of all accessory genes (Supplementary Figures 4D, E). However, the dependence between host-wise annotation and phylogeny was not straightforward (Figure 3C). For instance, clade 2 encompassed primarily environmental samples, including two genomes isolated from insects and two strains found in plants, and was enriched with the respective specificity factors, yet, including one clinical isolate. Clades 1 and 7 contained primarily isolates lacking host annotations and were poor with specificity factors, and this was especially notable for clade 1 (Figures 3A, B). Clades 3 and 4–6 were presented mostly by human-associated strains. In spite of this, clade 4 encompassed one strain residing on plants, whereas clade 5 – insect-infecting isolate. Notably, the respective distribution of virulence determinants was uniform and independent from the phylogeny (Figure 3D).

After that, we used the CoinFinder tool to dissect the co-occurrence of specificity factors. The co-occurrence graph contained 12 connected components with the first harboring soft-core genes and the others containing shell genes with 59 assemblies minimum (80% of presence). Only one human-related factor (sugar efflux transporter) formed a connected component with RNA chaperone/antiterminator CspA and isochorismatase family protein (Supplementary Table 11). The absence of host-wise co-occurrence networks could be caused by a limited number of genomes; thus further large-scale studies may reveal more associations. Furthermore, this observation might also be explained by the richness of dispensable pangenome components.

### 3.5. Functional annotation of gene clusters

To reveal functional features of gene groups, we performed group-wise enrichment analysis and compared pangenome orthologous clusters (core, accessory, and unique) and virulence determinants (core and accessory) with host-wise specificity factors. On the whole, the number of annotations, for each group, was equal to the number of genes in the clusters, except for those containing paralogs which gained a higher number of functional terms (Supplementary Figure 5). There were 85 clusters in which some genes were annotated with others having no annotation terms (Supplementary Table 12). When considering the absolute number of terms per gene cluster, several distinct lines were found corresponding to the mean number of annotation terms per gene (Supplementary Figure 6). However, within GO (Gene ontology) system, a more dispersed distribution with high variance for core genes was found (Supplementary Figure 6). Notably, 99.8% of gene clusters shared identical sets of functional terms for each gene within the cluster (Supplementary Figure 7). Those that were non-identical mostly lacked annotations for certain genes. Core genes, both pan-genomic and core virulence, contained more clusters with functional terms, especially when considering COG (Cluster of Orthologous Genes) and KEGG (Kyoto Encyclopedia of Genes and Genomes) systems (Supplementary Figure 8). Human-associated specificity factors incorporated slightly more annotated clusters than those related to plant and insect infections, respectively (Supplementary Figure 8). Interestingly, clusters with incomplete annotations were likely involved in processes affecting virulence, e.g., cell motility, ion binding, two-component system activity, enteric infections, etc. (Supplementary Figures 9A–E; Supplementary Table 13). We thus might assume that missed data in the existing databases could be supplemented using terms from pangenome clusters.

**FIGURE 4:**
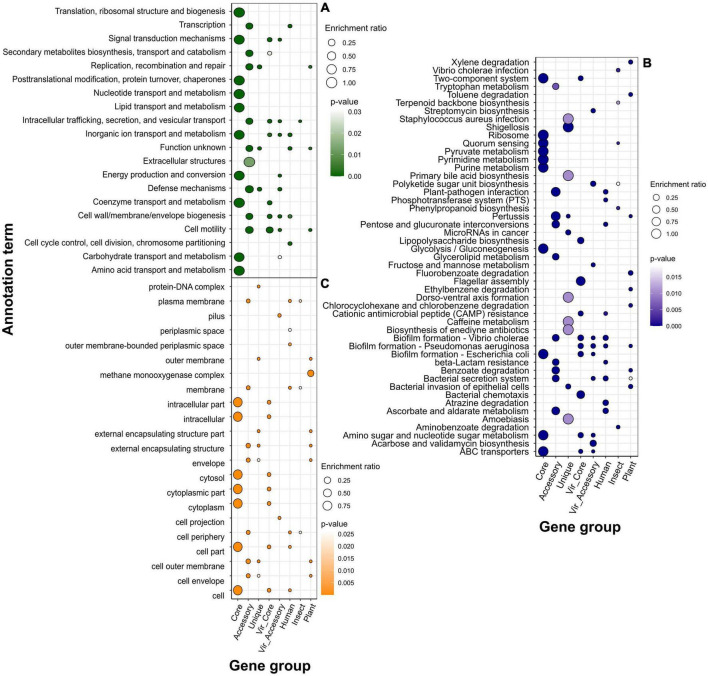
Over-represented annotation terms in different gene groups, namely, pangenomic (core, accessory, and unique), virulence (core and accessory), and specificity genes attributed to a particular host (human, insect, plant) using COG (Cluster of Orthologous Genes) **(A)**, KEGG (Kyoto Encyclopedia of Genes and Genomes) **(B)**, and GO (Gene Ontology) cellular component **(C)** annotation systems. The color denotes adjusted *p*-values, and the dot size depicts the enrichment ratio.

Within the COG system, core genes were enriched with primary cellular and metabolically processes’ categories, while accessory genes, in their turn, were linked with extracellular structures, secondary metabolites’ synthesis, motility, cell wall biogenesis, replication and recombination, and defense mechanisms, which was a characteristic of virulence determinants (both core and accessory) as well (Figure 4A; Supplementary Tables 14, 15). Human-associated genes incorporated categories linked with transcription and cell cycle control/division. Infecting insects was related to intracellular trafficking and secretion, whereas preference toward plants implied the same enrichments as those reported for unique genes, i.e., replication and recombination, and defense mechanisms.

Over-representation of KEGG terms was consistent with the COG enrichments showing core genes to participate in base cell and metabolic processes (glycolysis, transport, translation, etc.), and accessory component to antibiotic resistance, secretion, glycerolipid metabolism, and, surprisingly, the plant-pathogen interaction which was present in human-associated but not plant-related gene set (Figure 4B). Unique genes show pathways related to enteric infections and invasion of epithelial cells. The gene group delineating infection in humans was functionally similar virulence factors, such as biofilm formation, ABC transporters, two-component system, lipopolysaccharide biosynthesis, cationic antimicrobial peptide (CAMP) resistance, secretion, and polyketide synthesis. Other annotations related to this set of genes were the phosphotransferase system and resistance to beta-lactams. The main functional hallmarks of assemblies isolated from insects are connected to secondary metabolites’ synthesis, including terpenoids, polyketides, and phenylpropanoids. Apart from terms within the accessory genes, samples collected from plants were enriched with the degradation of chemical substances such as xylene, toluene, and fluorobenzoate.

**FIGURE 5:**
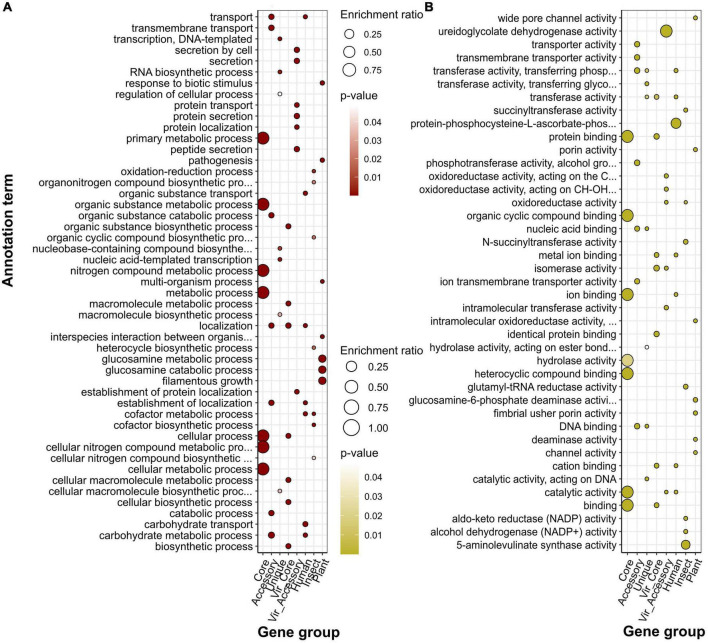
Over-represented annotation terms in different gene groups, namely, pangenomic (core, accessory, and unique), virulence (core and accessory), and specificity genes attributed to a particular host (human, insect, plant) using GO (Gene Ontology) biological processes **(A)** and molecular function **(B)** ontologies. The color denotes adjusted *p*-values, and the dot size depicts the enrichment ratio.

Speaking of predicted localization from the GO cellular component system, core, as well as core virulence genes’ products, are localized in intracellular compartments, while proteins encoded by accessory genes, and specificity factors associated with humans and insects reside in cellular membranes, periplasmic space, and cell periphery (Figure 4C). The group of genes determining plant infection shared the same terms as singletons (extracellular capsule and outer membrane). Enrichments within the biological processes category for general pangenome components and virulence genes all pertained to different primary cellular and metabolic pathways, including nitrogen compounds carbohydrate metabolism, transport transcription, and regulation (Figure 5A). In contrast, determinants of preference toward hosts displayed more specific annotations as follows: cofactor metabolic processes (humans), oxidation-reduction, organic cyclic, and heterocyclic compounds metabolism (insects), interspecies interaction, pathogenesis, response to biotic stimulus, filamentous growth, and glucosamine metabolism (plants). Fitting into the general frame, molecular functions of the products encoded by core genes encompassed binging of various chemical moieties including proteins, ions, and organic compounds, while the accessory component and known virulence determinants are engaged in the transport and transferring of phosphorous groups and DNA binging thus implying their possible role in signal transduction and transcriptional regulation (Figure 5B). In addition to terms shared with core genes and virulence factors (binding and transferase activity), group-specific characteristics of specificity determinants linked with human infection included phosphoryl transfer-driven membrane transport. Molecular functions of genes attributed to insect hosts represented a spectrum of metabolic reactions governed by diverse enzymes such as succinyltransferase, aldo-keto reductase, 5-aminolevulinate synthase, and others. Finally, over-represented terms encompassed by plant-associated genes were porins and channels, deaminase, and oxidoreductase activities.

With the aim to find functional similarity between host specificity factors, we used all significant enrichments of six gene groups: three host-wise accessory genes and Scoary-reported associations by performing the k-means clustering procedure (Supplementary Table 16). The elbow method revealed that four clusters were optimal to be used (Supplementary Figures 10A–D). All clusterizations were convergent and provided the same grouping patterns (Supplementary Figures 11A–D). The largest cluster united host-wise accessory components, whilst human-, insect-, and plant-associated specificity determinants fell into distinct clusters. It thus could be proposed that genomes of strains isolated from different hosts are functionally similar *per se*, however, specificity determinants show varying functional pathways.

### 3.6. Mobile genetic elements associated with virulence specificity

**FIGURE 6:**
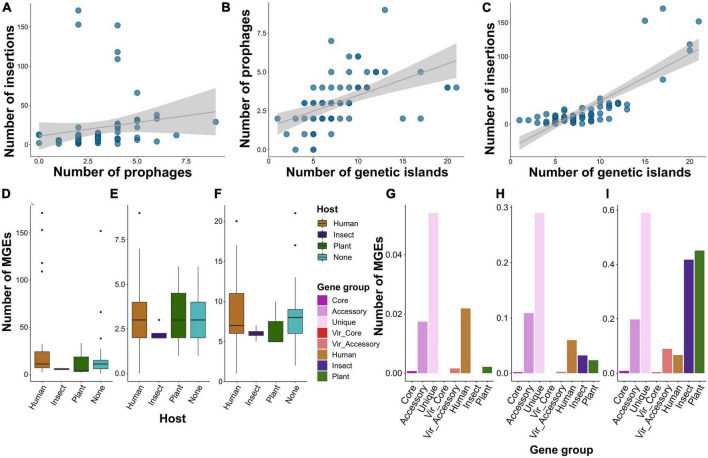
Shown are the features of mobile genetic elements (MGEs) presented in *S. marcescens* genomes. **(A)** Dependence between the number of insertions and phages, genetic islands and phages **(B)**, insertions and genetic islands **(C)**. Each dot represents an assembly, and the gray line denotes linear relationships between the abundance of MGEs. **(D)** The number of insertions, phages **(E)**, and genetic islands **(F)** among *S. marcescens* genomes grouped by the host attributed to the respective assemblies. **(G)** The percentage of genes within gene groups, namely, pangenomic (core, accessory, and unique), virulence (core and accessory), and specificity genes attributed to a particular host (human, insect, plant), located within the sequences of insertions, phages **(H)**, and genetic islands **(I)**.

To find possible connections between virulence and specificity with mobile genetic elements (MGEs) we searched for these elements in the genomic dataset used. We considered three types of MGEs: insertion sequences (ISs), prophages, and genetic islands (GIs). We found a positive correlation between the number of the elements, however, significant associations according to the Pearson test were reported for prophages vs. GIs (*p*-value < 6.5e-06) and ISs vs. GIs (*p*-value < 2.2e-16) with the corresponding correlation coefficients of 0.5 and 0.8 (Figures 6A–C; Supplementary Table 17). Median values of MGEs’ abundance reached 10, 3, and 7 for ISs, prophages, and GIs, respectively. When considering the assemblies grouped by the attributed host, we found that genomes isolated from plants and insects bore half as many ISs as other assemblies, while the mean frequency of prophages and GIs were comparable and close to overall median estimates (Figures 6D–F).

Having shown no remarkable difference between the absolute number of the MGEs, we decided to evaluate the percentage of genes from the above-defined gene groups whose genomic coordinates intersected with the elements (Figures 6G–I). Of note, the smallest percentage was shown for core and virulence genes with the exception of accessory virulence factors with circa 10% of genes located within prophages. Accessory and especially unique genes were most prominently linked with MGEs with almost 60% of singletons residing in GIs. Insertions contained the lowest number of genes. Nevertheless, 2% of human-associated genes and 4% of singletons were located within insertions. The most conspicuous feature of specificity factors attributed to insect and plant infections was a high proportion (more than 40%) of genetic islands harboring them. Next, we verified how many of the host-wise specificity genes were located within MGEs (Supplementary Table 18). Human-attributed assemblies contained 96 insertions bearing 1 to 2 genes. Furthermore, we found 13 prophages with more than 18 factors in human-infecting strains. Two assemblies isolated from plants were enriched with genomic islands. One of these (GCF_001417865.2) harbored tree GIs with 69, 37, and 28 specificity determinants, and the other (GCF_011602465.1)–one island bearing 15 genes. Finally, we identified two genomic islands with 41 insect-associated genes both in two assemblies, namely GCF_013122155.1 and GCF_015160915.1.

## 4. Discussion

Due to the severe impact of *S. marcescens*-induced nosocomial infections, current studies focus on revealing phylogenetic lineages of clinical isolates and/or identifying antibiotic-resistance genes (Moradigaravand et al., 2016; Ono et al., 2022). By contrast, our work aims to explore *S. marcescens* adaptations to certain hosts, namely, humans, insects, and plants using a pangenome-wide associating method (pan-GWAS) which was applied by Ono et al. (2022) when revealing accessory genes specifically conserved in two large clinical and hospital-associated *S. marcescens* clades. Nonetheless, to the best of our knowledge, a host-wise pangenomic exploration of putative specificity factors has not been done yet. Abreo and Altier (2019) conducted the research with relatively similar goals regarding plant growth promoting (PGP) and virulence traits, yet the authors used a pre-existing list of virulence (22) and PGP (21) genes, whilst concerning that this approach could leave unknown genetic traits hidden within the genomes.

We analyzed 73 *S. marcescens* genomes with a complete level of assembly and performed a methodological comparison to choose the most optimal pipeline. Circa 76% of the species’ pangenome belonged to the accessory (Figure 2C), which matched with other topical research items in which comparable values were obtained (Moradigaravand et al., 2016; Abreo and Altier, 2019; Ono et al., 2022; Williams et al., 2022). These inferences coupled with an open pangenome, first explicitly estimated by us, imply considerable genetic variability of *S*. *marcescens* strains and, therefore, might explain such a broad host range and multiple activities. Another indication of genetic plasticity was a high proportion of mobile diverse MGEs bearing accessory genes (Figures 6G–I). It is worth noting, that the abundance of MGEs *per se* is not indicative of the preferred host (Figures 6D–F). That being said, human-isolated samples contained plenty of prophages, and the list of specificity factors included many phage tail and baseplate assembly proteins. That is of importance as many phages associated with virulent strains encode extracellular toxins, effector proteins participating in invasion (Fortier and Sekulovic, 2013). Plant- and insect-associated factors, by contrast, tend to be located on GIs. Their role in shaping the genome of *S*. *marcescens* was linked to the emergence of MDR (multidrug-resistant) phenotypes (Mataseje et al., 2014; Gambino et al., 2021). Of particular note, *Serratia* sp. SCBI was found to harbor unique GI related to enteric infections of *Caenorhabditis briggsae* (Abebe-Akele et al., 2015). Henceforth, it is not the quantity but the quality of MGEs that probably delineates adaptation to a particular host.

We identified eight human-associated specificity factors with significant adjusted *p*-value. Of these, MgtC/SapB was found to induce biofilm formation of *Pseudomonas aeruginosa* (Redfern et al., 2021). The YgdI/YgdR lipoprotein was also found in the biofilm matrix proteome from a *P. aeruginosa* clinical isolate (Egorova et al., 2022). LysR family proteins are recognized as well-known regulators of multiple *S. marcescens* activities, including cell motility, exopolysaccharide synthesis, and biofilm formation (Pan et al., 2020). On top of that, these transcription factors account for the increased virulence of bacterial pathogens, e.g., *P. aeruginosa* (Yeung et al., 2009) and *Vibrio cholera* (Bogard et al., 2012). Finally, examinations of diverse bacterial pathogens’ virulence mechanisms revealed the essential role of ABC transporters (Garmory and Titball, 2004). Among other proteins encoded by genes related to human infection according to empirical *p*-values, we identified ankyrin repeat domain-containing protein which could possibly manipulate hosts functions (Al-Khodor et al., 2010), and porins which are considered effectors of infection (Achouak et al., 2001). Top phenome-wide inferences successfully collate with the most notable functional enrichments such as transport, including iron acquisition, and periplasmic space. Periplasmic proteins (Liu et al., 2009; Moreira et al., 2013) and the process of ion acquisition (Payne, 1993; Rodriguez and Smith, 2006) are considered essential in managing bacterial pathogenesis.

Three top-scored putative specificity factors linked with insect infections were GNAT (Gcn5-related N-acetyltransferase), cysteine hydrolase, and isochorismatase, corroborating common functional traits attributed to metabolic pathways, namely, oxidases, reductases, transferases, and dehydrogenases. GNAT proteins acetylate different substrates and play a role in bacterial antibiotic resistance (Czub et al., 2018). Arylalkylamine N-acetyltransferase (aaNAT) belonging to the GNAT family has received attention due to its involvement in regulating the synthesis of neuromediators in insects (Tang et al., 2022). Hence, it could be hypothesized *S. marcescens* pathogenesis in insects may well be carried out via neurotoxicity. Zoopathogenic bacteria also utilize cysteine hydrolase to impair host immune responses (Kȩdzior et al., 2016). Another mechanism to modulate the behavior of host cells lies in triggering the autophagy of host cells provoked by isochorismatase activity (Wang et al., 2016).

Selected putative contributors to host preference toward plants fell into two categories: virulence and PGP agents. The first category encompassed the MurR/RpiR family transcriptional regulator, the homolog of which was demonstrated to regulate cell wall synthesis (Jaeger and Mayer, 2008). It is quite probable that these alterations can lead to evading plant recognition and immunity via the reduction of host perception of cell wall peptidoglycans (Gust, 2015). We also found multiple pan-GWAS signals corresponding to the type VI secretion system, a key infection mediator of plant pathogens (Rainey, 1999). The second category comprised oxalurate catabolism protein HpxZ and amino acid ABC transporter permease. Oxalurate metabolism constitutes a branched complex network of chemical transformations ending in a release of ammonium ions, which alleviate plants’ tolerance during nitrogen deficiency (Izaguirre-Mayoral et al., 2018). As for amino acid transporters, they were shown to participate in plants’ dialogue with rhizospheric bacteria, e.g., *Pseudomonas fluorescens* (Bernal et al., 2018). Like with other hosts, plant-associated specificity determinants corresponded to a functional annotation with enrichments related to porins, cell wall and encapsulating structures, and interspecies interactions. The functional feature that deserves particular attention is xenobiotic catabolism. Being probiotic bacteria, some strains of *S. marcescens* ensure plant resistance to chemical pollutants due to their capability of neutralizing benzo(a)pyrene (Kotoky and Pandey, 2020), fluorobenzoate (Zhang et al., 2020), toluene and other toxic moieties (Stancu, 2016).

We also examined how the revealed specificity factors are distributed within the genomes analyzed regarding phylogenetic relationships between them (Figures 3D, E). Of the subclades selected, clade 2 represented four strains reported to be pathogenic against insects and plants, respectively. This clade was poor in human-associated factors but enriched with those attributed to plants and insects. The clade contained two entomopathogenic isolates, namely strain FY and Byron invading intestines *Drosophila melanogaster* (Liu et al., 2020), and *Curculio caryae* (Wu et al., 2021), respectively. Several strains constituting this clade were included in the analysis performed by Abreo and Altier (2019) as a part of the environmental clade named 1c encompassing one strain with PGP properties and lacking clinical isolates. This strain was absent in our analysis as we collected only complete level assemblies. However, the clade contained one presumably plant-pathogenic sample B3R3 shown to be close to the *S. marcescens* strain causing leaf spot disease on industrial hemp (*Cannabis sativa* L.) (Schappe et al., 2019) and BP2 strain found in seeds of *Jatropha curcas* with unknown activity. It must be noted that strain U36365 which fell into this phylogenetic group was described as resistant to antibiotic therapy causative agent of urinary tract infection (Sahni et al., 2016). Inexplicably, the isolate formed green-colored but not red-pigmented colonies (Sahni et al., 2016). Clades 3 and 6 contained almost exclusively human-infecting clinical samples, whereas clades 1 and 7 were presented by infectious to human strains coupled with assemblies of unknown origin. Clades 4 and 5 were of intriguing composition. Even though they contained primarily human-associated genomes, PGPB strain RSC-14 pertained to clade 4 (Khan et al., 2017), and strain N10A28, claimed to be a pathogen of *Apis mellifera*, – to clade 5. Another remarkable observation that we found was the distribution of insect-related specificity factors along clades from 2 to 5, inclusively, since 7 assemblies were enriched with these determinants comparable with those infecting insects. To this set of genomes belonged 2 isolates of environmental origin, namely rhizosphere soil and water surface of an oligotrophic pond, seed-residing BP2 strain, three human pathogens, and one genome of unknown origin. It hence may be proposed that these genomes may possess hidden insecticidal activity.

We should note that a relatively high portion of strains not attributed to any host has led to several important caveats, decreasing the number of statistically significant associations. Moreover, there is an obvious skew toward studies related to pathogenic clinical isolates which could lower the statistical power of the inferences. We deliberately included assemblies with unassigned hosts, thereby making the sample as close as possible to the general population instead of cherry-picking based on predetermined properties of interest. Nonetheless, the methodology used provided us with certain novel genetic determinants, underlining its high sensitivity.

## 5. Conclusion

Although belonging to the *Enterobacteriaceae* family, *S. marcescens* is able to colonize not only the mammalian intestine but a wide range of hosts, including invertebrates and even plants. Such specialization to different ecological niches raises the question regarding the diversity of molecular factors, which determine the host specificity. Having studied the pangenome of a relatively limited but high-quality and complete set of genomes, we have found significant variability in virulence factors, with only housekeeping genes forming the core part. By analyzing the reconstructed pangenome, we identified novel factors that possibly determine the adaptation to the particular group of hosts. Of note, patterns of functional enrichments allowed us to hypothesize that the respective host preference is carried out through distinct molecular mechanisms of virulence. Moreover, the found candidates only scarcely intersect with the known virulence factors presented in the VFDB resource. Not only do specific genes delineate host adaptation but also *S. marcescens* isolates attributed to different hosts harbor group-specific mobile elements, which provides insights into possible ways of how the specificity factors are spread through bacterial populations. To sum up, our methodology helped us to reveal new factors delineating host specificity on the basis of the relatively small dataset with scarce metadata, which is quite common in such studies. The found incongruence between the distributions and the abundance of host specificity determinants and known virulence factors might imply that virulence itself does not delineate adaptations to particular hosts.

## Data availability statement

The original contributions presented in this study are included in the article/Supplementary material, further inquiries can be directed to the corresponding author.

## Author contributions

AS performed pangenome and phylogeny reconstruction, created scripts, visualized the results, and wrote and revised the original manuscript. AM performed pan-GWAS and validated the results. IS contributed to text editing. AN contributed to text editing and writing, project administration, and funding. KA conceptualized and supervised the study and wrote and revised the manuscript. All authors read and approved the final manuscript.

## Footnotes

1
https://github.com/rrwick/Assembly-Dereplicator

2
https://rdrr.io/cran/shipunov/

3
http://geneontology.org/

## References

[B1] Abebe-Akele F. Tisa L. S. Cooper V. S. Hatcher P. J. Abebe E. Thomas W. K. (2015). Genome sequence and comparative analysis of a putative entomopathogenic *Serratia* isolated from *Caenorhabditis briggsae*. *BMC Genomics* 16:531. 10.1186/s12864-015-1697-8 26187596 PMC4506600

[B2] Abreo E. Altier N. (2019). Pangenome of *Serratia marcescens* strains from nosocomial and environmental origins reveals different populations and the links between them. *Sci. Rep.* 9:46. 10.1038/s41598-018-37118-0 30631083 PMC6328595

[B3] Achouak W. Heulin T. Pagès J.-M. (2001). Multiple facets of bacterial porins. *FEMS Microbiol. Lett.* 199 1–7. 10.1111/j.1574-6968.2001.tb10642.x 11356559

[B4] Alexa A. Rahnenfuhrer J. (2022). *topGO: Enrichment analysis for gene ontology. R package version 2.48.0.*

[B5] Al-Khodor S. Price C. T. Kalia A. Abu Kwaik Y. (2010). Functional diversity of ankyrin repeats in microbial proteins. *Trends Microbiol.* 18 132–139. 10.1016/j.tim.2009.11.004 19962898 PMC2834824

[B6] Ashburner M. Ball C. A. Blake J. A. Botstein D. Butler H. Cherry J. M. (2000). Gene ontology: Tool for the unification of biology. The Gene Ontology Consortium. *Nat. Genet.* 25 25–29. 10.1038/75556 10802651 PMC3037419

[B7] Barrett T. Clark K. Gevorgyan R. Gorelenkov V. Gribov E. Karsch-Mizrachi I. (2012). BioProject and BioSample databases at NCBI: Facilitating capture and organization of metadata. *Nucleic Acids Res.* 40 D57–D63. 10.1093/nar/gkr1163 22139929 PMC3245069

[B8] Baya A. M. Toranzo A. E. Lupiani B. Santos Y. Hetrick F. M. (1992). *Serratia marcescens*: A potential pathogen for fish. *J. Fish Dis.* 15 15–26. 10.1111/j.1365-2761.1992.tb00632.x

[B9] Bernal P. Llamas M. A. Filloux A. (2018). Type VI secretion systems in plant-associated bacteria. *Environ. Microbiol.* 20 1–15. 10.1111/1462-2920.13956 29027348 PMC5813230

[B10] Bertelli C. Brinkman F. S. L. (2018). Improved genomic island predictions with IslandPath-DIMOB. *Bioinformatics* 34 2161–2167. 10.1093/bioinformatics/bty095 29905770 PMC6022643

[B11] Besler K. R. Little E. L. (2017). Diversity of *Serratia marcescens* strains associated with cucurbit yellow vine disease in Georgia. *Plant Dis.* 101 129–136. 10.1094/PDIS-05-16-0618-RE 30682311

[B12] Bogard R. W. Davies B. W. Mekalanos J. J. (2012). MetR-regulated *Vibrio cholerae* metabolism is required for virulence. *MBio* 3:e00236-12. 10.1128/mBio.00236-12 23015737 PMC3448163

[B13] Brynildsrud O. Bohlin J. Scheffer L. Eldholm V. (2016). Rapid scoring of genes in microbial pan-genome-wide association studies with Scoary. *Genome Biol.* 17:238. 10.1186/s13059-016-1108-8 27887642 PMC5124306

[B14] Chen L. Yang J. Yu J. Yao Z. Sun L. Shen Y. (2005). VFDB: A reference database for bacterial virulence factors. *Nucleic Acids Res.* 33 D325–D328. 10.1093/nar/gki008 15608208 PMC539962

[B15] Chen X. Hitchings M. D. Mendoza J. E. Balanza V. Facey P. D. Dyson P. J. (2017). Comparative genomics of facultative bacterial symbionts isolated from European *Orius* species reveals an ancestral symbiotic association. *Front. Microbiol.* 8:1969. 10.3389/fmicb.2017.01969 29067021 PMC5641365

[B16] Chung W.-C. Chen L.-L. Lo W.-S. Kuo P.-A. Tu J. Kuo C.-H. (2013). Complete genome sequence of *Serratia marcescens* WW4. *Genome Announc.* 1:e0012613. 10.1128/genomeA.00126-13 23558532 PMC3622982

[B17] Cock P. J. A. Antao T. Chang J. T. Chapman B. A. Cox C. J. Dalke A. (2009). Biopython: Freely available Python tools for computational molecular biology and bioinformatics. *Bioinformatics* 25 1422–1423. 10.1093/bioinformatics/btp163 19304878 PMC2682512

[B18] Czub M. P. Zhang B. Chiarelli M. P. Majorek K. A. Joe L. Porebski P. J. (2018). A Gcn5-related N-acetyltransferase (GNAT) capable of acetylating polymyxin B and colistin antibiotics *in vitro*. *Biochemistry* 57 7011–7020. 10.1021/acs.biochem.8b00946 30499668 PMC6474815

[B19] Darriba D. Posada D. Kozlov A. M. Stamatakis A. Morel B. Flouri T. (2020). ModelTest-NG: A new and scalable tool for the selection of DNA and protein evolutionary models. *Mol. Biol. Evol.* 37 291–294. 10.1093/molbev/msz189 31432070 PMC6984357

[B20] Demidenko E. (2018). The next-generation K-means algorithm. *Stat. Anal. Data Min.* 11 153–166. 10.1002/sam.11379 30073045 PMC6062903

[B21] Dessì A. Puddu M. Testa M. Marcialis M. A. Pintus M. C. Fanos V. (2009). *Serratia marcescens* infections and outbreaks in neonatal intensive care units. *J. Chemother.* 21 493–499. 10.1179/joc.2009.21.5.493 19933039

[B22] Ding W. Baumdicker F. Neher R. A. (2018). panX: Pan-genome analysis and exploration. *Nucleic Acids Res.* 46:e5. 10.1093/nar/gkx977 29077859 PMC5758898

[B23] Dupriez F. Rejasse A. Rios A. Lefebvre T. Nielsen-LeRoux C. (2022). Impact and persistence of *Serratia marcescens* in *Tenebrio molitor* larvae and feed under optimal and stressed mass rearing conditions. *Insects* 13:458. 10.3390/insects13050458 35621793 PMC9148086

[B24] Egorova D. A. Solovyev A. I. Polyakov N. B. Danilova K. V. Scherbakova A. A. Kravtsov I. N. (2022). Biofilm matrix proteome of clinical strain of *P. aeruginosa* isolated from bronchoalveolar lavage of patient in intensive care unit. *Microb. Pathog.* 170:105714. 10.1016/j.micpath.2022.105714 35973647

[B25] Escribano E. Saralegui C. Moles L. Montes M. T. Alba C. Alarcón T. (2019). Influence of a *Serratia marcescens* outbreak on the gut microbiota establishment process in low-weight preterm neonates. *PLoS One* 14:e0216581. 10.1371/journal.pone.0216581 31112570 PMC6529157

[B26] Fodor A. Palkovics L. Végh A. (2022). First report of *Serratia marcescens* from oleander in Hungary. *Phytopathol. Mediterr.* 61 311–317. 10.36253/phyto-13354 37408125

[B27] Fortier L.-C. Sekulovic O. (2013). Importance of prophages to evolution and virulence of bacterial pathogens. *Virulence* 4 354–365. 10.4161/viru.24498 23611873 PMC3714127

[B28] Friman M. J. Eklund M. H. Pitkälä A. H. Rajala-Schultz P. J. Rantala M. H. J. (2019). Description of two *Serratia marcescens* associated mastitis outbreaks in Finnish dairy farms and a review of literature. *Acta Vet. Scand.* 61:54. 10.1186/s13028-019-0488-7 31727124 PMC6857314

[B29] Gambino A. S. Déraspe M. Álvarez V. E. Quiroga M. P. Corbeil J. Roy P. H. (2021). *Serratia marcescens* SCH909 as reservoir and source of genetic elements related to wide dissemination of antimicrobial resistance mechanisms. *FEMS Microbiol. Lett.* 368:fnab086. 10.1093/femsle/fnab086 34264334

[B30] Garmory H. S. Titball R. W. (2004). ATP-binding cassette transporters are targets for the development of antibacterial vaccines and therapies. *Infect. Immun.* 72 6757–6763. 10.1128/IAI.72.12.6757-6763.2004 15557595 PMC529116

[B31] Gillis A. Rodríguez M. Santana M. A. (2014). *Serratia marcescens* associated with bell pepper (*Capsicum annuum* L.) soft-rot disease under greenhouse conditions. *Eur. J. Plant Pathol.* 138 1–8. 10.1007/s10658-013-0300-x

[B32] Gosselin S. Fullmer M. S. Feng Y. Gogarten J. P. (2022). Improving phylogenies based on average nucleotide identity, incorporating saturation correction and nonparametric bootstrap support. *Syst. Biol.* 71 396–409. 10.1093/sysbio/syab060 34289044 PMC8830074

[B33] Gust A. A. (2015). Peptidoglycan perception in plants. *PLoS Pathog.* 11:e1005275. 10.1371/journal.ppat.1005275 26679352 PMC4683077

[B34] Haddy R. I. Mann B. L. Nadkarni D. D. Cruz R. F. Elshoff D. J. Buendia F. C. (1996). Nosocomial infection in the community hospital: Severe infection due to *Serratia* species. *J. Fam. Pract.* 42 273–277. 8636679

[B35] Hasan M. F. Islam M. A. Sikdar B. (2020). First report of *Serratia marcescens* associated with black rot of *Citrus sinensis* fruit, and evaluation of its biological control measures in Bangladesh. *F1000Res.* 9:1371. 10.12688/f1000research.27657.2 34804504 PMC8581594

[B36] Huerta-Cepas J. Forslund K. Coelho L. P. Szklarczyk D. Jensen L. J. von Mering C. (2017). Fast genome-wide functional annotation through orthology assignment by eggNOG-Mapper. *Mol. Biol. Evol.* 34 2115–2122. 10.1093/molbev/msx148 28460117 PMC5850834

[B37] Izaguirre-Mayoral M. L. Lazarovits G. Baral B. (2018). Ureide metabolism in plant-associated bacteria: Purine plant-bacteria interactive scenarios under nitrogen deficiency. *Plant Soil* 428 1–34. 10.1007/s11104-018-3674-x

[B38] Jaeger T. Mayer C. (2008). The transcriptional factors MurR and catabolite activator protein regulate N-acetylmuramic acid catabolism in *Escherichia coli*. *J. Bacteriol.* 190 6598–6608. 10.1128/JB.00642-08 18723630 PMC2566201

[B39] Kamata R. Matsumoto K. Okamura R. Yamamoto T. Maeda H. (1985). The serratial 56K protease as a major pathogenic factor in serratial keratitis. Clinical and experimental study. *Ophthalmology* 92 1452–1459. 10.1016/s0161-6420(85)33855-1 3906492

[B40] Kanehisa M. Goto S. (2000). KEGG: Kyoto encyclopedia of genes and genomes. *Nucleic Acids Res.* 28 27–30. 10.1093/nar/28.1.27 10592173 PMC102409

[B41] Katoh K. Standley D. M. (2013). MAFFT multiple sequence alignment software version 7: Improvements in performance and usability. *Mol. Biol. Evol.* 30 772–780. 10.1093/molbev/mst010 23329690 PMC3603318

[B42] Kȩdzior M. Seredyński R. Gutowicz J. (2016). Microbial inhibitors of cysteine proteases. *Med. Microbiol. Immunol.* 205 275–296. 10.1007/s00430-016-0454-1 27048482

[B43] Khan A. R. Park G.-S. Asaf S. Hong S.-J. Jung B. K. Shin J.-H. (2017). Complete genome analysis of *Serratia marcescens* RSC-14: A plant growth-promoting bacterium that alleviates cadmium stress in host plants. *PLoS One* 12:e0171534. 10.1371/journal.pone.0171534 28187139 PMC5302809

[B44] Kitts P. A. Church D. M. Thibaud-Nissen F. Choi J. Hem V. Sapojnikov V. (2016). Assembly: A resource for assembled genomes at NCBI. *Nucleic Acids Res.* 44 D73–D80. 10.1093/nar/gkv1226 26578580 PMC4702866

[B45] Kotoky R. Pandey P. (2020). Rhizosphere assisted biodegradation of benzo(a)pyrene by cadmium resistant plant-probiotic *Serratia marcescens* S2I7, and its genomic traits. *Sci. Rep.* 10:5279. 10.1038/s41598-020-62285-4 32210346 PMC7093395

[B46] Kozlov A. M. Darriba D. Flouri T. Morel B. Stamatakis A. (2019). RAxML-NG: A fast, scalable and user-friendly tool for maximum likelihood phylogenetic inference. *Bioinformatics* 35 4453–4455. 10.1093/bioinformatics/btz305 31070718 PMC6821337

[B47] Kurz C. L. Chauvet S. Andrès E. Aurouze M. Vallet I. Michel G. P. F. (2003). Virulence factors of the human opportunistic pathogen *Serratia marcescens* identified by in vivo screening. *EMBO J.* 22 1451–1460. 10.1093/emboj/cdg159 12660152 PMC152903

[B48] Lancaster L. J. (1962). Role of *Serratia* species in urinary tract infections. *Arch. Intern. Med.* 109 536–539. 10.1001/archinte.1962.03620170034005 14461900

[B49] Létoffé S. Ghigo J. M. Wandersman C. (1994). Iron acquisition from heme and hemoglobin by a *Serratia marcescens* extracellular protein. *Proc. Natl. Acad. Sci. U.S.A.* 91 9876–9880. 10.1073/pnas.91.21.9876 7937909 PMC44920

[B50] Li P. Kwok A. H. Y. Jiang J. Ran T. Xu D. Wang W. (2015). Comparative genome analyses of *Serratia marcescens* FS14 reveals its high antagonistic potential. *PLoS One* 10:e0123061. 10.1371/journal.pone.0123061 25856195 PMC4391916

[B51] Liu L. Tan S. Jun W. Smith A. Meng J. Bhagwat A. A. (2009). Osmoregulated periplasmic glucans are needed for competitive growth and biofilm formation by *Salmonella enterica* serovar Typhimurium in leafy-green vegetable wash waters and colonization in mice. *FEMS Microbiol. Lett.* 292 13–20. 10.1111/j.1574-6968.2008.01462.x 19222578

[B52] Liu W. Kang R. Lim K. L. Tan E. K. (2020). Complete genome sequence of *Serratia marcescens* FY, isolated from *Drosophila melanogaster*. *Microbiol. Resour. Announc.* 9:e00755-20. 10.1128/MRA.00755-20 33153999 PMC7645654

[B53] Löytynoja A. (2014). Phylogeny-aware alignment with PRANK. *Methods Mol. Biol.* 1079 155–170. 10.1007/978-1-62703-646-7_10 24170401

[B54] Mahlen S. D. (2011). *Serratia infections*: From military experiments to current practice. *Clin. Microbiol. Rev.* 24 755–791. 10.1128/CMR.00017-11 21976608 PMC3194826

[B55] Mataseje L. F. Boyd D. A. Delport J. Hoang L. Imperial M. Lefebvre B. (2014). *Serratia marcescens* harbouring SME-type class A carbapenemases in Canada and the presence of blaSME on a novel genomic island, SmarGI1-1. *J. Antimicrob. Chemother.* 69 1825–1829. 10.1093/jac/dku040 24659751

[B56] Matteoli F. P. Passarelli-Araujo H. Reis R. J. A. da Rocha L. O. de Souza E. M. Aravind L. (2018). Genome sequencing and assessment of plant growth-promoting properties of a *Serratia marcescens* strain isolated from vermicompost. *BMC Genomics* 19:750. 10.1186/s12864-018-5130-y 30326830 PMC6192313

[B57] Matteoli F. P. Pedrosa-Silva F. Dutra-Silva L. Giachini A. J. (2021). The global population structure and beta-lactamase repertoire of the opportunistic pathogen *Serratia marcescens*. *Genomics* 113 3523–3532. 10.1016/j.ygeno.2021.08.009 34400240

[B58] Merlino C. P. (1924). Bartolomeo Bizio’s Letter to the most eminent priest, Angelo Bellani, concerning the phenomenon of the red colored polenta. *J. Bacteriol.* 9 527–543. 10.1128/jb.9.6.527-543.1924 16559067 PMC379088

[B59] Mir A. Rotger L. Rosselló F. (2018). Sound Colless-like balance indices for multifurcating trees. *PLoS One* 13:e0203401. 10.1371/journal.pone.0203401 30252858 PMC6155497

[B60] Moradigaravand D. Boinett C. J. Martin V. Peacock S. J. Parkhill J. (2016). Recent independent emergence of multiple multidrug-resistant *Serratia marcescens* clones within the United Kingdom and Ireland. *Genome Res.* 26 1101–1109. 10.1101/gr.205245.116 27432456 PMC4971767

[B61] Moreira C. G. Herrera C. M. Needham B. D. Parker C. T. Libby S. J. Fang F. C. (2013). Virulence and stress-related periplasmic protein (VisP) in bacterial/host associations. *Proc. Natl. Acad. Sci. U.S.A.* 110 1470–1475. 10.1073/pnas.1215416110 23302685 PMC3557018

[B62] O’Leary N. A. Wright M. W. Brister J. R. Ciufo S. Haddad D. McVeigh R. (2016). Reference sequence (RefSeq) database at NCBI: Current status, taxonomic expansion, and functional annotation. *Nucleic Acids Res.* 44 D733–D745. 10.1093/nar/gkv1189 26553804 PMC4702849

[B63] Ondov B. D. Treangen T. J. Melsted P. Mallonee A. B. Bergman N. H. Koren S. (2016). Mash: Fast genome and metagenome distance estimation using MinHash. *Genome Biol.* 17:132. 10.1186/s13059-016-0997-x 27323842 PMC4915045

[B64] Ono T. Taniguchi I. Nakamura K. Nagano D. S. Nishida R. Gotoh Y. (2022). Global population structure of the *Serratia marcescens* complex and identification of hospital-adapted lineages in the complex. *Microb. Genomics* 8:000793. 10.1099/mgen.0.000793 35315751 PMC9176281

[B65] Page A. J. Cummins C. A. Hunt M. Wong V. K. Reuter S. Holden M. T. G. (2015). Roary: Rapid large-scale prokaryote pan genome analysis. *Bioinformatics* 31 3691–3693. 10.1093/bioinformatics/btv421 26198102 PMC4817141

[B66] Page A. J. Taylor B. Delaney A. J. Soares J. Seemann T. Keane J. A. (2016). SNP-sites: Rapid efficient extraction of SNPs from multi-FASTA alignments. *Microb. Genomics* 2:e000056. 10.1099/mgen.0.000056 28348851 PMC5320690

[B67] Pan X. Sun C. Tang M. You J. Osire T. Zhao Y. (2020). LysR-Type transcriptional regulator MetR controls prodigiosin production, methionine biosynthesis, cell motility, H(2)O(2) tolerance, heat tolerance, and exopolysaccharide synthesis in *Serratia marcescens*. *Appl. Environ. Microbiol.* 86:e02241-19. 10.1128/AEM.02241-19 31791952 PMC6997736

[B68] Payne S. M. (1993). Iron acquisition in microbial pathogenesis. *Trends Microbiol.* 1 66–69. 10.1016/0966-842x(93)90036-q 8044465

[B69] Price M. N. Dehal P. S. Arkin A. P. (2010). FastTree 2 – approximately Maximum-Likelihood trees for large alignments. *PLoS One* 5:e9490. 10.1371/journal.pone.0009490 20224823 PMC2835736

[B70] Pye G. W. Jacobson E. R. Newell S. M. Scase T. Heard D. J. Dennis P. M. (1999). *Serratia marcescens* infection in a gopher tortoise, *Gopherus polyphemus*, and use of magnetic resonance imaging in diagnosing systemic disease. *Bull. Assoc. Reptil. Amphib. Vet.* 9 8–11. 10.5818/1076-3139.9.4.8

[B71] Quinlan A. R. Hall I. M. (2010). BEDTools: A flexible suite of utilities for comparing genomic features. *Bioinformatics* 26 841–842. 10.1093/bioinformatics/btq033 20110278 PMC2832824

[B72] Rainey P. B. (1999). Adaptation of *Pseudomonas fluorescens* to the plant rhizosphere. *Environ. Microbiol.* 1 243–257. 10.1046/j.1462-2920.1999.00040.x 11207743

[B73] Redfern J. Wallace J. van Belkum A. Jaillard M. Whittard E. Ragupathy R. (2021). Biofilm associated genotypes of multiple antibiotic resistant *Pseudomonas aeruginosa*. *BMC Genomics* 22:572. 10.1186/s12864-021-07818-5 34311706 PMC8314537

[B74] Rocha E. P. C. Danchin A. (2002). Base composition bias might result from competition for metabolic resources. *Trends Genet.* 18 291–294. 10.1016/S0168-9525(02)02690-2 12044357

[B75] Rodriguez G. M. Smith I. (2006). Identification of an ABC transporter required for iron acquisition and virulence in *Mycobacterium tuberculosis*. *J. Bacteriol.* 188 424–430. 10.1128/JB.188.2.424-430.2006 16385031 PMC1347291

[B76] Sahni R. D. Amalanathan R. Devanga Ragupathi N. K. Mathai J. Veeraraghavan B. Biswas I. (2016). Complete genome sequence of *Serratia marcescens* U36365, a green pigment-producing strain isolated from a patient with urinary tract infection. *Genome Announc.* 4:e00837-16. 10.1128/genomeA.00837-16 27516523 PMC4982302

[B77] Saidenberg A. B. S. Teixeira R. H. F. Astolfi-Ferreira C. S. Knöbl T. Ferreira A. J. P. (2007). *Serratia marcescens* infection in a swallow-tailed hummingbird. *J. Wildl. Dis.* 43 107–110. 10.7589/0090-3558-43.1.107 17347399

[B78] Sand A. Holt M. K. Johansen J. Brodal G. S. Mailund T. Pedersen C. N. S. (2014). tqDist: A library for computing the quartet and triplet distances between binary or general trees. *Bioinformatics* 30 2079–2080. 10.1093/bioinformatics/btu157 24651968

[B79] Saralegui C. Ponce-Alonso M. Pérez-Viso B. Moles Alegre L. Escribano E. Lázaro-Perona F. (2020). Genomics of *Serratia marcescens* isolates causing outbreaks in the same pediatric unit 47 years apart: Position in an updated phylogeny of the species. *Front. Microbiol.* 11:451. 10.3389/fmicb.2020.00451 32296400 PMC7136904

[B80] Schappe T. Ritchie D. F. Thiessen L. D. (2019). First report of *Serratia marcescens* causing a leaf spot disease on industrial hemp (*Cannabis sativa*). *Plant Dis.* 104:1248. 10.1094/PDIS-04-19-0782-PDN

[B81] Shikov A. E. Malovichko Y. V. Nizhnikov A. A. Antonets K. S. (2022). Current methods for recombination detection in bacteria. *Int. J. Mol. Sci.* 23:6257. 10.3390/ijms23116257 35682936 PMC9181119

[B82] Shimuta K. Ohnishi M. Iyoda S. Gotoh N. Koizumi N. Watanabe H. (2009). The hemolytic and cytolytic activities of *Serratia marcescens* phospholipase A (PhlA) depend on lysophospholipid production by PhlA. *BMC Microbiol.* 9:261. 10.1186/1471-2180-9-261 20003541 PMC2800117

[B83] Sikorowski P. P. Lawrence A. M. Inglis G. D. (2001). Effects of *Serratia marcescens* on rearing of the tobacco budworm (Lepidoptera: *Noctuidae*). *Am. Entomol.* 47 51–60. 10.1093/ae/47.1.51

[B84] Simão F. A. Waterhouse R. M. Ioannidis P. Kriventseva E. V. Zdobnov E. M. (2015). BUSCO: Assessing genome assembly and annotation completeness with single-copy orthologs. *Bioinformatics* 31 3210–3212. 10.1093/bioinformatics/btv351 26059717

[B85] Snipen L. Liland K. H. (2015). micropan: An R-package for microbial pan-genomics. *BMC Bioinformatics* 16:79. 10.1186/s12859-015-0517-0 25888166 PMC4375852

[B86] Stam E. (2002). Does imbalance in phylogenies reflect only bias? *Evolution* 56 1292–1295. 10.1111/j.0014-3820.2002.tb01440.x 12144028

[B87] Stancu M. M. (2016). Response mechanisms in *Serratia marcescens* IBB(Po15) during organic solvents exposure. *Curr. Microbiol.* 73 755–765. 10.1007/s00284-016-1108-7 27538581

[B88] Starikova E. V. Tikhonova P. O. Prianichnikov N. A. Rands C. M. Zdobnov E. M. Ilina E. N. (2020). Phigaro: High-throughput prophage sequence annotation. *Bioinformatics* 36 3882–3884. 10.1093/bioinformatics/btaa250 32311023

[B89] Steinegger M. Söding J. (2017). MMseqs2 enables sensitive protein sequence searching for the analysis of massive data sets. *Nat. Biotechnol.* 35 1026–1028. 10.1038/nbt.3988 29035372

[B90] Suryawanshi R. K. Patil C. D. Borase H. P. Narkhede C. P. Salunke B. K. Patil S. V. (2015). Mosquito larvicidal and pupaecidal potential of prodigiosin from *Serratia marcescens* and understanding its mechanism of action. *Pestic. Biochem. Physiol.* 123 49–55. 10.1016/j.pestbp.2015.01.018 26267052

[B91] Tang Y. Chen H. Lin Z. Zhang L. Upadhyay A. Liao C. (2022). Evolutionary genomics analysis reveals gene expansion and functional diversity of arylalkylamine N-acetyltransferases in the *Culicinae* subfamily of mosquitoes. *Insect Sci.* 30 569–581. 10.1111/1744-7917.13100 35922881

[B92] Tang Y. Horikoshi M. Li W. (2016). ggfortify: Unified interface to visualize statistical results of popular R packages. *R J.* 8 478–489. 10.32614/RJ-2016-060

[B93] Tatusov R. L. Galperin M. Y. Natale D. A. Koonin E. V. (2000). The COG database: A tool for genome-scale analysis of protein functions and evolution. *Nucleic Acids Res.* 28 33–36. 10.1093/nar/28.1.33 10592175 PMC102395

[B94] Tettelin H. Riley D. Cattuto C. Medini D. (2008). Comparative genomics: The bacterial pan-genome. *Curr. Opin. Microbiol.* 11 472–477. 10.1016/j.mib.2008.09.006 19086349

[B95] Tonkin-Hill G. MacAlasdair N. Ruis C. Weimann A. Horesh G. Lees J. A. (2020). Producing polished prokaryotic pangenomes with the Panaroo pipeline. *Genome Biol.* 21:180. 10.1186/s13059-020-02090-4 32698896 PMC7376924

[B96] Tripura C. Sashidhar B. Podile A. R. (2007). Ethyl methanesulfonate mutagenesis–enhanced mineral phosphate solubilization by groundnut-associated *Serratia marcescens* GPS-5. *Curr. Microbiol.* 54 79–84. 10.1007/s00284-005-0334-1 17200805

[B97] Vaikuntapu P. R. Rambabu S. Madhuprakash J. Podile A. R. (2016). A new chitinase-D from a plant growth promoting *Serratia marcescens* GPS5 for enzymatic conversion of chitin. *Bioresour. Technol.* 220 200–207. 10.1016/j.biortech.2016.08.055 27567481

[B98] Wang Y. Zhang K. Shi X. Wang C. Wang F. Fan J. (2016). Critical role of bacterial isochorismatase in the autophagic process induced by *Acinetobacter baumannii* in mammalian cells. *FASEB J.* 30 3563–3577. 10.1096/fj.201500019R 27432399 PMC5024702

[B99] Whelan F. J. Hall R. J. McInerney J. O. (2021). Evidence for selection in the abundant accessory gene content of a prokaryote pangenome. *Mol. Biol. Evol.* 38 3697–3708. 10.1093/molbev/msab139 33963386 PMC8382901

[B100] Whelan F. J. Rusilowicz M. McInerney J. O. (2020). Coinfinder: Detecting significant associations and dissociations in pangenomes. *Microb. Genomics* 6:e000338. 10.1099/mgen.0.000338 32100706 PMC7200068

[B101] Wickham H. (2016). *ggplot2: Elegant graphics for data analysis.* New York, NY: Springer-Verlag. 10.1007/978-3-319-24277-4

[B102] Williams D. J. Grimont P. A. D. Cazares A. Grimont F. Ageron E. Pettigrew K. A. (2022). The genus *Serratia* revisited by genomics. *Nat. Commun.* 13:5195. 10.1038/s41467-022-32929-2 36057639 PMC9440931

[B103] Wu S. Blackburn M. B. Mizell R. F. Duncan L. W. Toews M. D. Sparks M. E. (2021). Novel associations in antibiosis stemming from an insect pupal cell. *J. Invertebr. Pathol.* 184:107655. 10.1016/j.jip.2021.107655 34411606

[B104] Xie Z. Tang H. (2017). ISEScan: Automated identification of insertion sequence elements in prokaryotic genomes. *Bioinformatics* 33 3340–3347. 10.1093/bioinformatics/btx433 29077810

[B105] Yeung A. T. Y. Torfs E. C. W. Jamshidi F. Bains M. Wiegand I. Hancock R. E. W. (2009). Swarming of *Pseudomonas aeruginosa* is controlled by a broad spectrum of transcriptional regulators, including MetR. *J. Bacteriol.* 191 5592–5602. 10.1128/JB.00157-09 19592586 PMC2737960

[B106] Zhang S. Chaluvadi S. R. Bennetzen J. L. (2020). Draft genome sequence of a *Serratia marcescens* strain isolated from the pitcher fluids of a *Sarracenia* pitcher plant. *Microbiol. Resour. Announc.* 9:e01216-19. 10.1128/MRA.01216-19 31919167 PMC6952653

[B107] Zhou Z. Charlesworth J. Achtman M. (2020). Accurate reconstruction of bacterial pan- and core genomes with PEPPAN. *Genome Res.* 30 1667–1679. 10.1101/gr.260828.120 33055096 PMC7605250

