# Supplementary material for: The man, the plant, and the insect: shooting host specificity determinants in Serratia marcescens pangenome
Source: Front Microbiol. 2023 Sep 12;14:1211999. doi: 10.3389/fmicb.2023.1211999 (PMC10656689; doi:10.3389/fmicb.2023.1211999)
Supplement: Supplementary file 1 [file Data_Sheet_1.docx]

Supplementary Material

The man, the plant, and the insect: shooting host specificity determinants in *Serratia marcescens* pangenome

**Anton E. Shikov, Anastasiya V. Merkushova, Iuliia A. Savina, Anton A. Nizhnikov, Kirill S. Antonets^*^**

*** Correspondence:** Kirill S. Antonets: k.antonets@arriam.ru

# Supplementary Figures and Tables

## Supplementary Figures

**
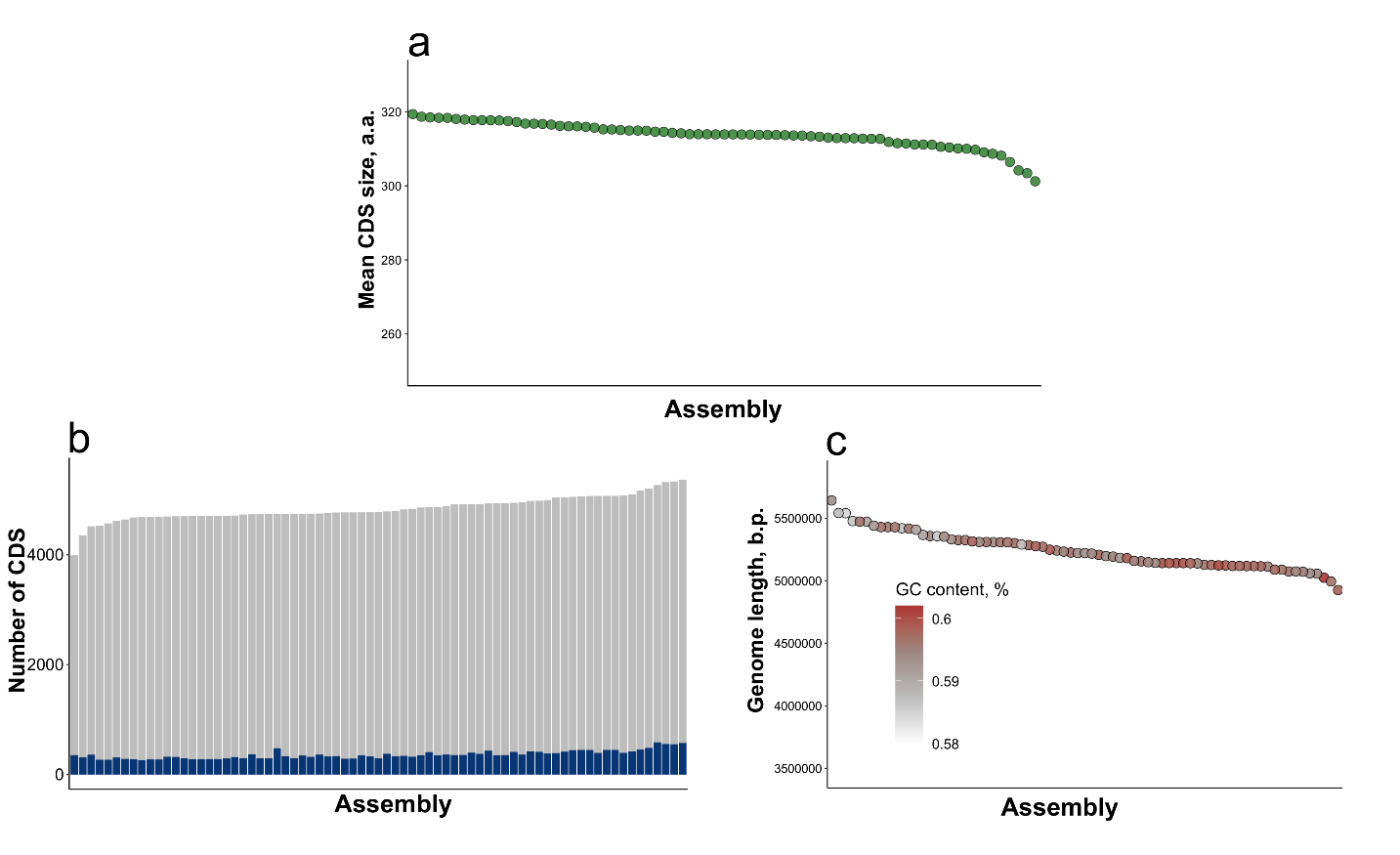
**

**Supplementary Figure S1.** Properties of analyzed *S. marcescens* genome assemblies (**a**) Mean assembly-wise CDS size represented as the number of amino acid residues per CDS. The assemblies are sorted by CDS size in ascending order. (**b**) The total number of CDS per assembly is shown as grey bars. Dark-blue bars illustrate the number of hypothetical proteins. (**c**) Total genome length of the assemblies used. The assemblies are sorted by total genome size in descending order. The intensity of the color codes GC content.


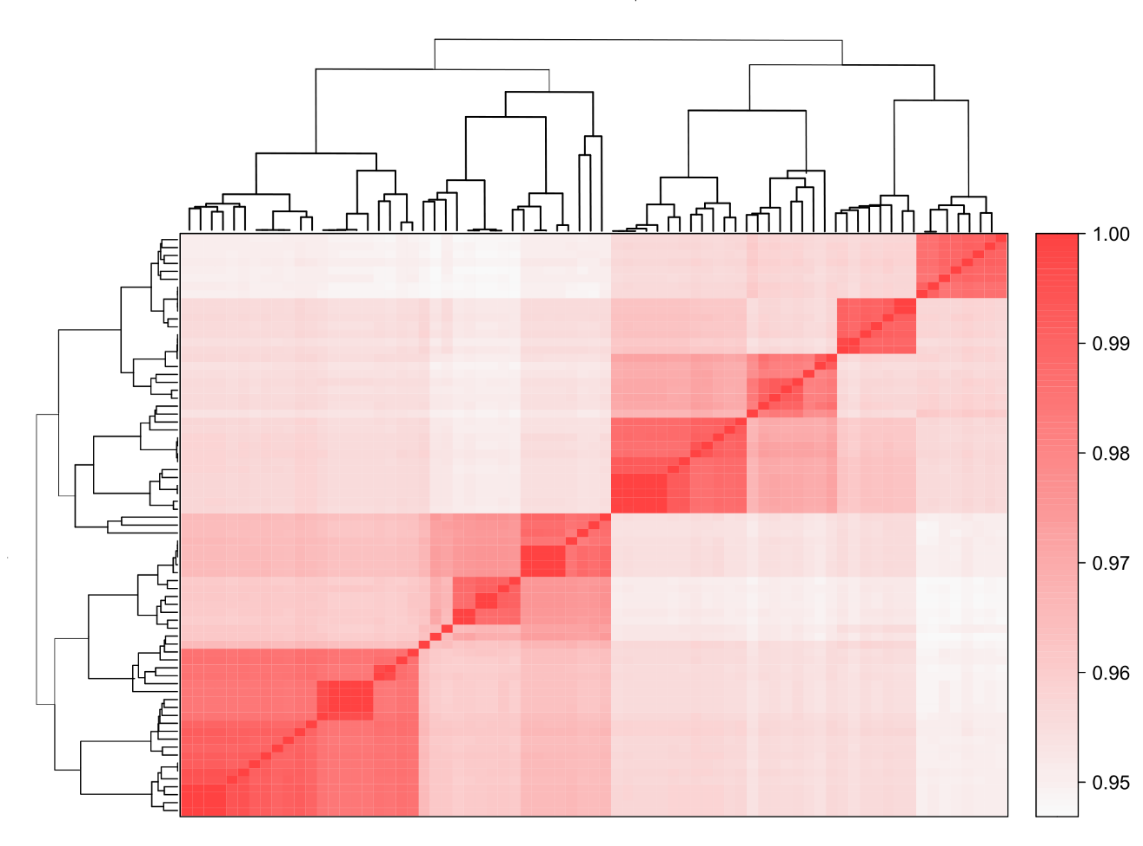


**Supplementary Figure S2.** Heatmap visualizing pair-wise genetic similarity between *S. marcescens* genomes. Rows and columns are ordered according to the adjacent tree obtained by the hierarchal clustering procedure. The intensity of the color is proportional to ANI (average nucleotide identity) values.


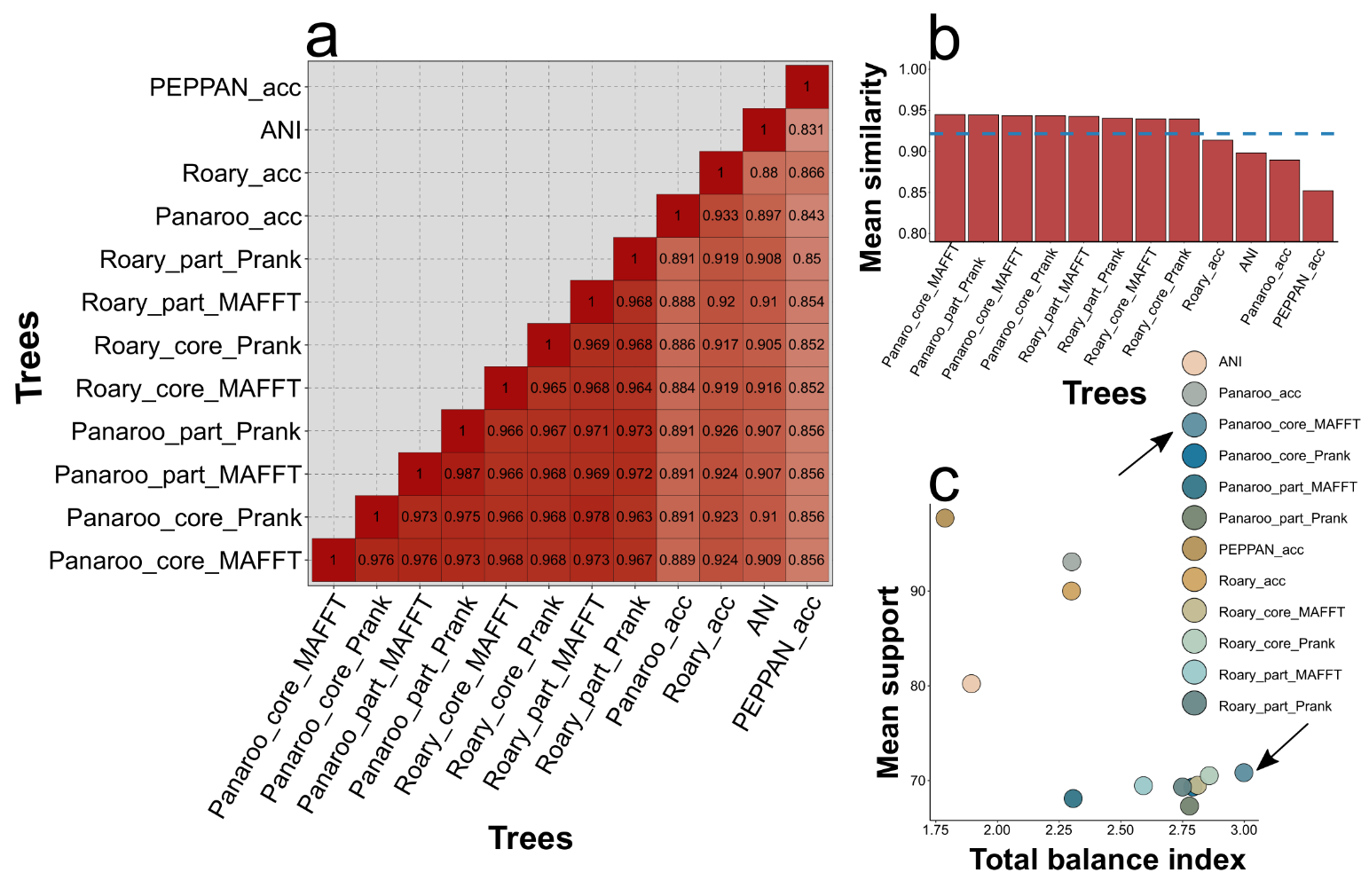


**Supplementary Figure S3.** Comparison of different phylogenetic approaches used on the set of *S. marcescens* genomes. Trees are based on pangenomes reconstructed with Panaroo, Roary, and PEPPAN. Names with the “*acc*” postfix denote phylogenies built on pseudo-alignments reflecting the presence/absence of accessory genes. Eight ML (maximum likelihood) trees obtained with RAxML-NG and based on core genes SNPs are encoded by labels with tree parts separated by underscores: the first encodes pangenomic tool, the second represents either concatenated alignment with a single evolutionary model (“_core_”) or partitioned alignment with individual evolutionary models per tree, and the third encodes the aligner used. Only two pangenomic programs were used due to the fact that PEPPAN does not provide core genome alignments. The ‘ANI’ label signifies a hierarchically clustered tree built on the distance matrix with pair-wise ANI (average nucleotide identity) values. (**a**) The topological similarity between the above-described phylogenies. The intensity of the color is proportional to similarity (1-quartet distance). (**b**) Mean topological similarity for trees. Plotted bars are mean similarity values for a certain tree between the set of all trees. The blue dotted line represents the overall mean. (**c**) Quality of the phylogenies built. Each point corresponds to a phylogenic tree. On the x-axis, the mean support of trees’ branches is shown. Trees with lower values are considered more balanced. Plotted on the y-axis is the normalized tree balance. The black arrow points to the chosen reference phylogeny.

**
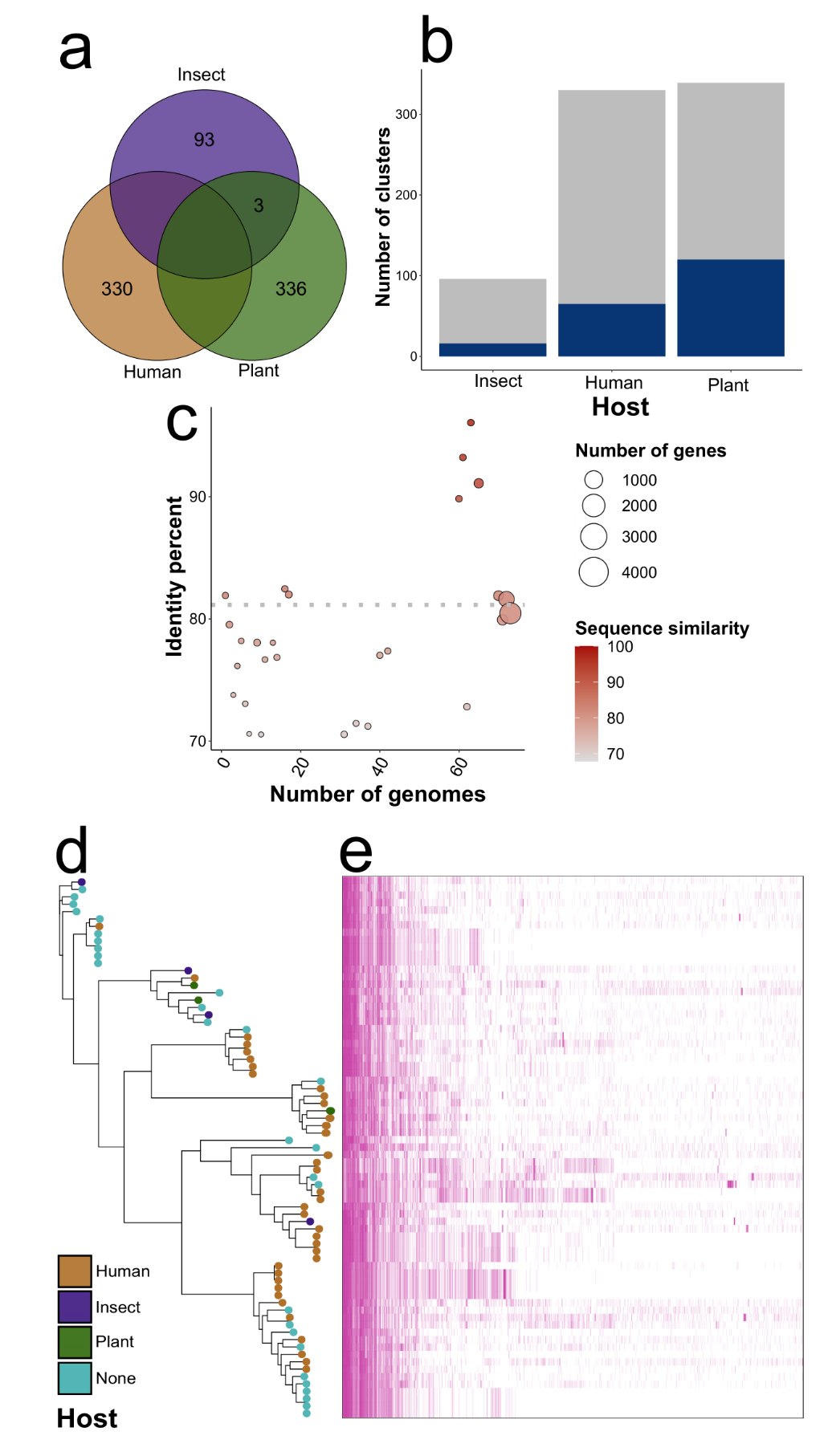
**

**Supplementary Figure S4.** Properties of specificity and virulence factors within *S. marcescens* genomes. (**a**) Venn diagram of shared and unique specificity markers revealed by Scoary. Shown is the pure output of the program. (**b**) Percentage of hypothetical proteins among the sets of specificity factors associated with a particular host. Grey bars represent the total number of markers, while the fraction of hypothetical proteins are denoted by dark blue bars. (**c**) Mean identity of virulence factors found in *S. marcescens* assemblies with the respective best hit from the VFDB (virulence factor database). The y-axis reflects the number of genomes in which a set of factors is found, while the y-axis and color intensity denote the mean identity percent for all hits in the respective group. The total number of factors is presented by the size of the dots. (**d**) The reference phylogeny of analyzed genomes reconstructed on the basis of core genome SNPs (single nucleotide polymorphisms) with leaves colored by the host. (**e**) The distribution of accessory genes within genomes ordered according to the adjacent phylogenetic tree.


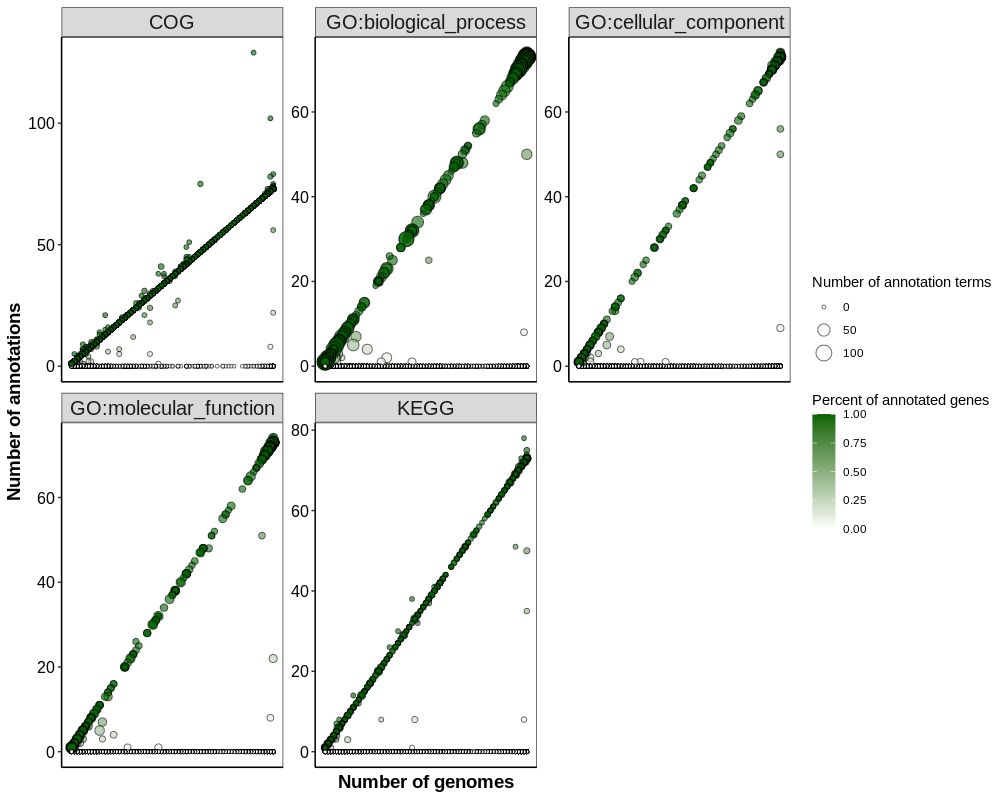


**Supplementary Figure S5.** The general functional landscape of pangenomic gene clusters of *S. marcescens* pangenome using COG (Cluster of Orthologous Genes), GO (Gene Ontology), and KEGG (Kyoto Encyclopedia of Genes and Genomes) annotation systems. Plotted on the x-axis is the number of genomes grouped within the gene cluster, and on the y-axis is the number of annotated genes (e.i., possessing at least one annotation). The color of the dots encodes the percentage of genes within the cluster with at least one annotation term attributed. The size of the dots represents the total abundance of annotation terms found in the gene cluster.


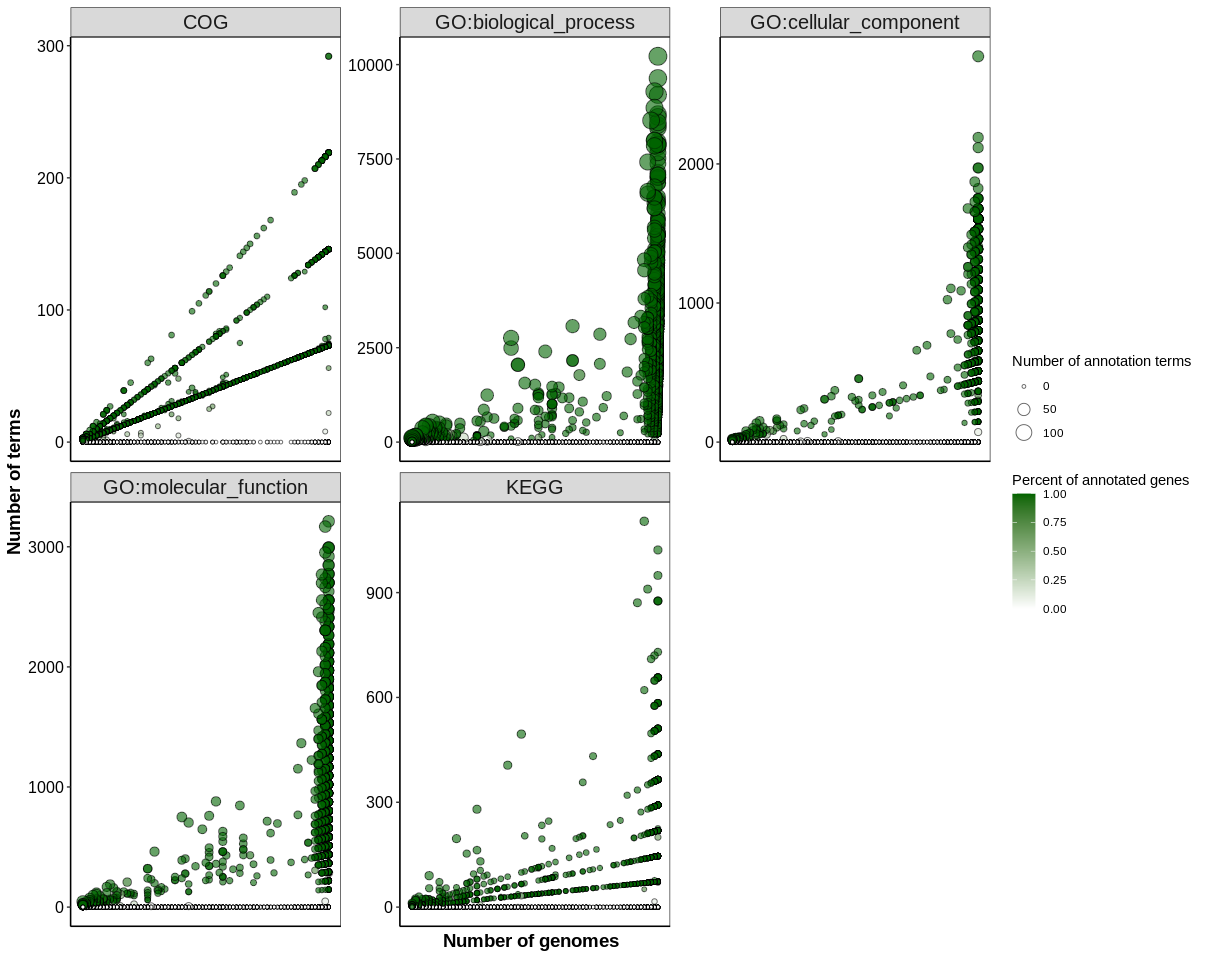


**Supplementary Figure S6.** The general functional landscape of pangenomic gene clusters of *S. marcescens* pangenome using COG (Cluster of Orthologous Genes), GO (Gene Ontology), and KEGG (Kyoto Encyclopedia of Genes and Genomes) annotation systems. Plotted on the x-axis is the number of genomes grouped within the gene cluster, and on the y-axis is the number of annotation terms. The color of the dots encodes the percentage of genes within the cluster with at least one annotation term attributed. The size of the dots represents the total abundance of annotation terms found in the gene cluster.


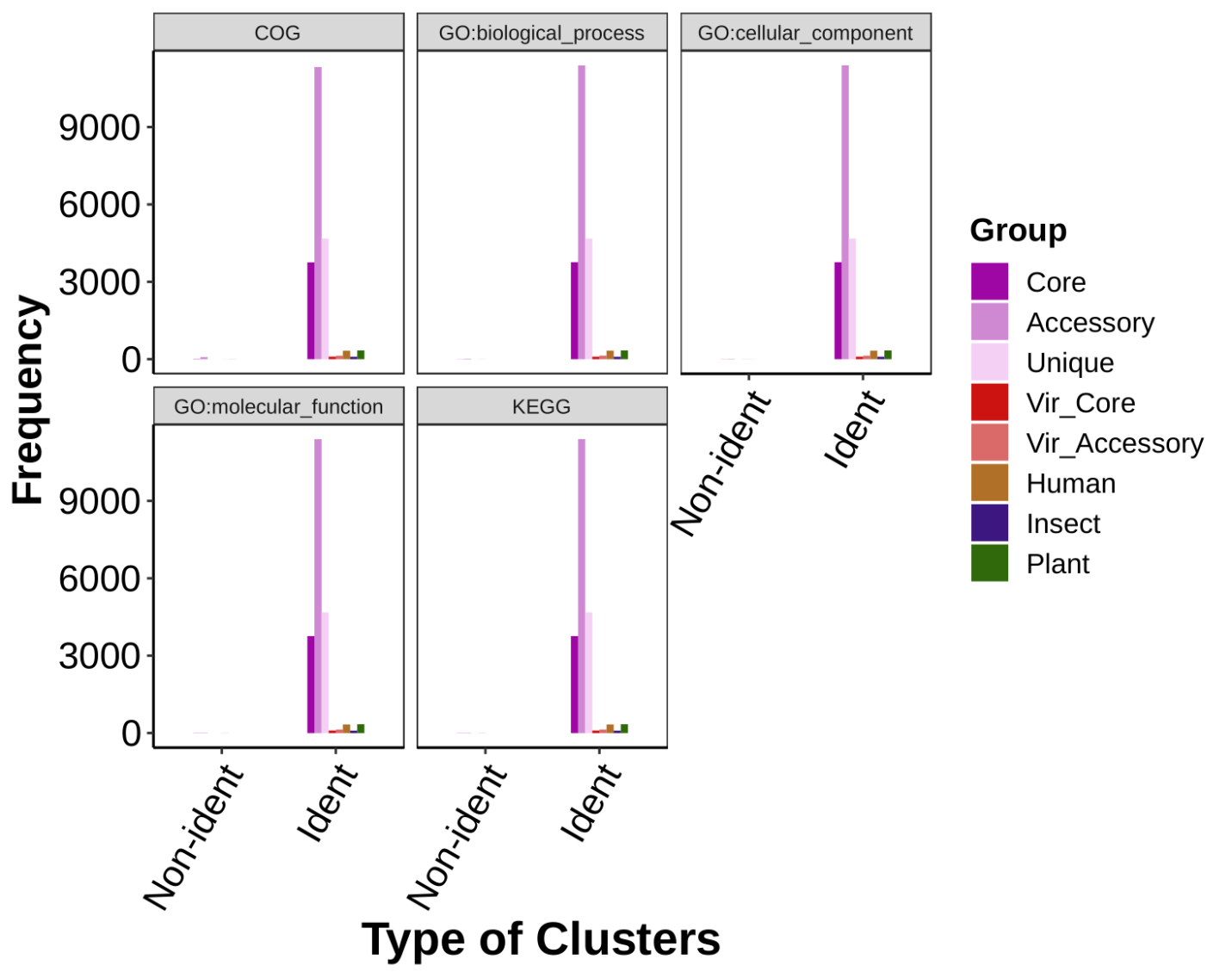


**Supplementary Figure S7.** The patterns of functional annotation of gene clusters within *S. marcescens* pangenome in different gene groups, namely, pangenomic (core, accessory, and unique), virulence (core and accessory), and specificity genes attributed to a particular host (human, insect, plant) using COG (Cluster of Orthologous Genes), GO (Gene Ontology), and KEGG (Kyoto Encyclopedia of Genes and Genomes) annotation systems. The frequency of two types of clusters is plotted, namely, those with identical annotation terms for all genes included in the cluster and those in which at least one annotation differs from others.


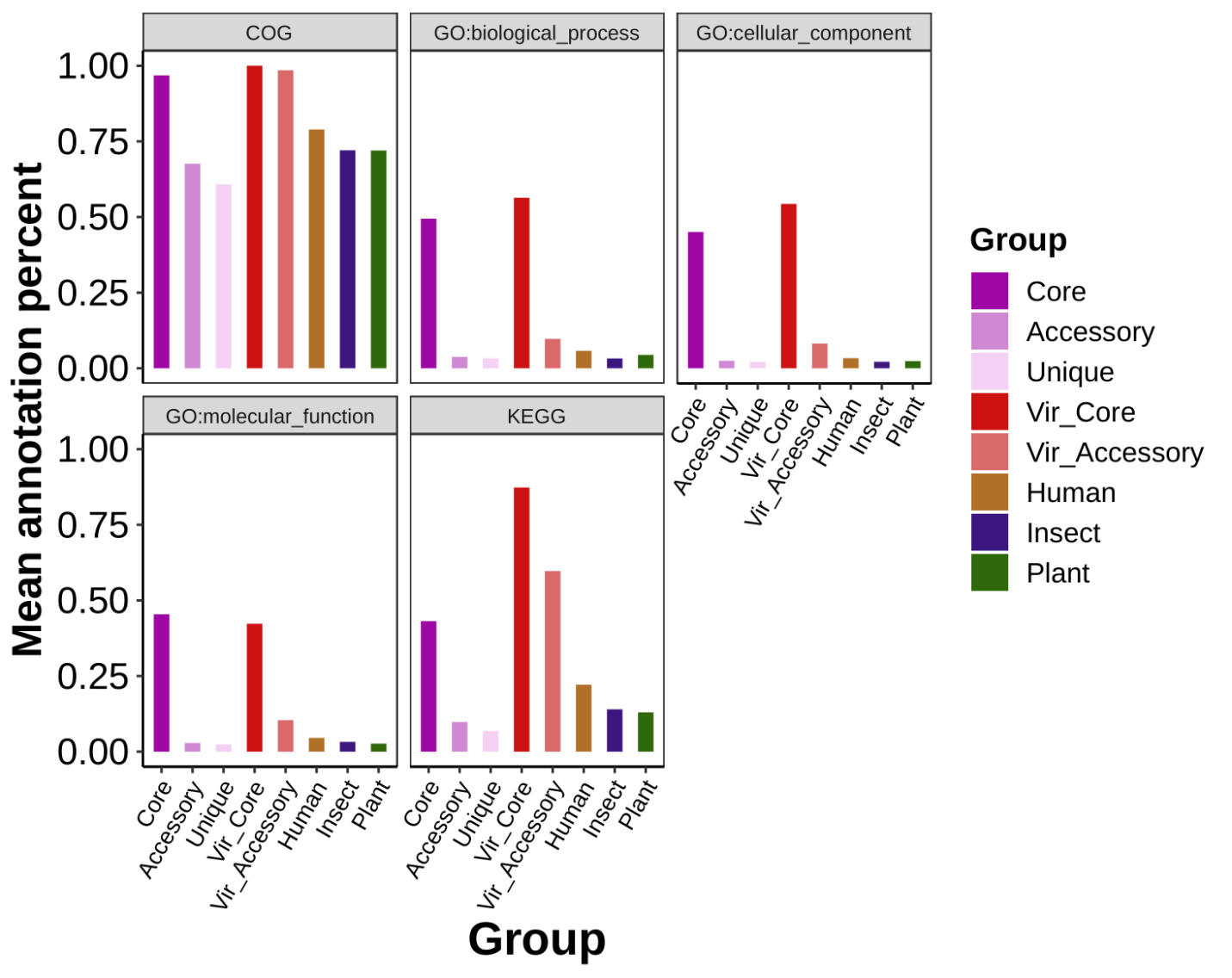


**Supplementary Figure S8.** The percentage of annotated within gene clusters of *S. marcescens* pangenome in different gene groups, namely, pangenomic (core, accessory, and unique), virulence (core and accessory), and specificity genes attributed to a particular host (human, insect, plant) using COG (Cluster of Orthologous Genes), GO (Gene Ontology), and KEGG (Kyoto Encyclopedia of Genes and Genomes) annotation systems.


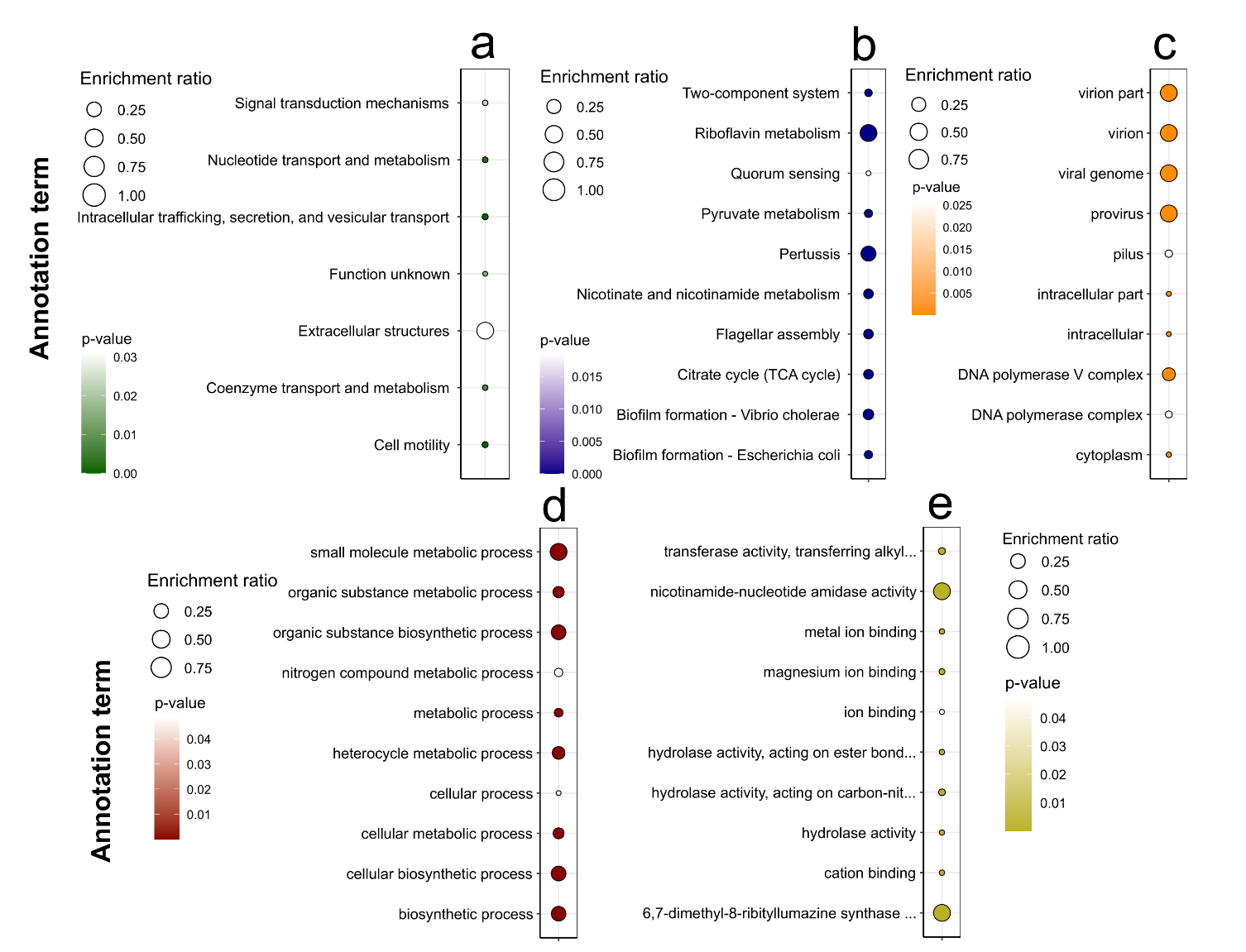


**Supplementary Figure S9.** Over-represented annotation terms of pangenomic clusters with non-identical annotations between genes using COG (Cluster of Orthologous Genes) (**a**), KEGG (Kyoto Encyclopedia of Genes and Genomes) (**b**), and GO (Gene Ontology) cellular component (**c**), biological processes (**d**) and molecular function (**e**) annotation systems. The color denotes adjusted p-values, and the dot size depicts the enrichment ratio.


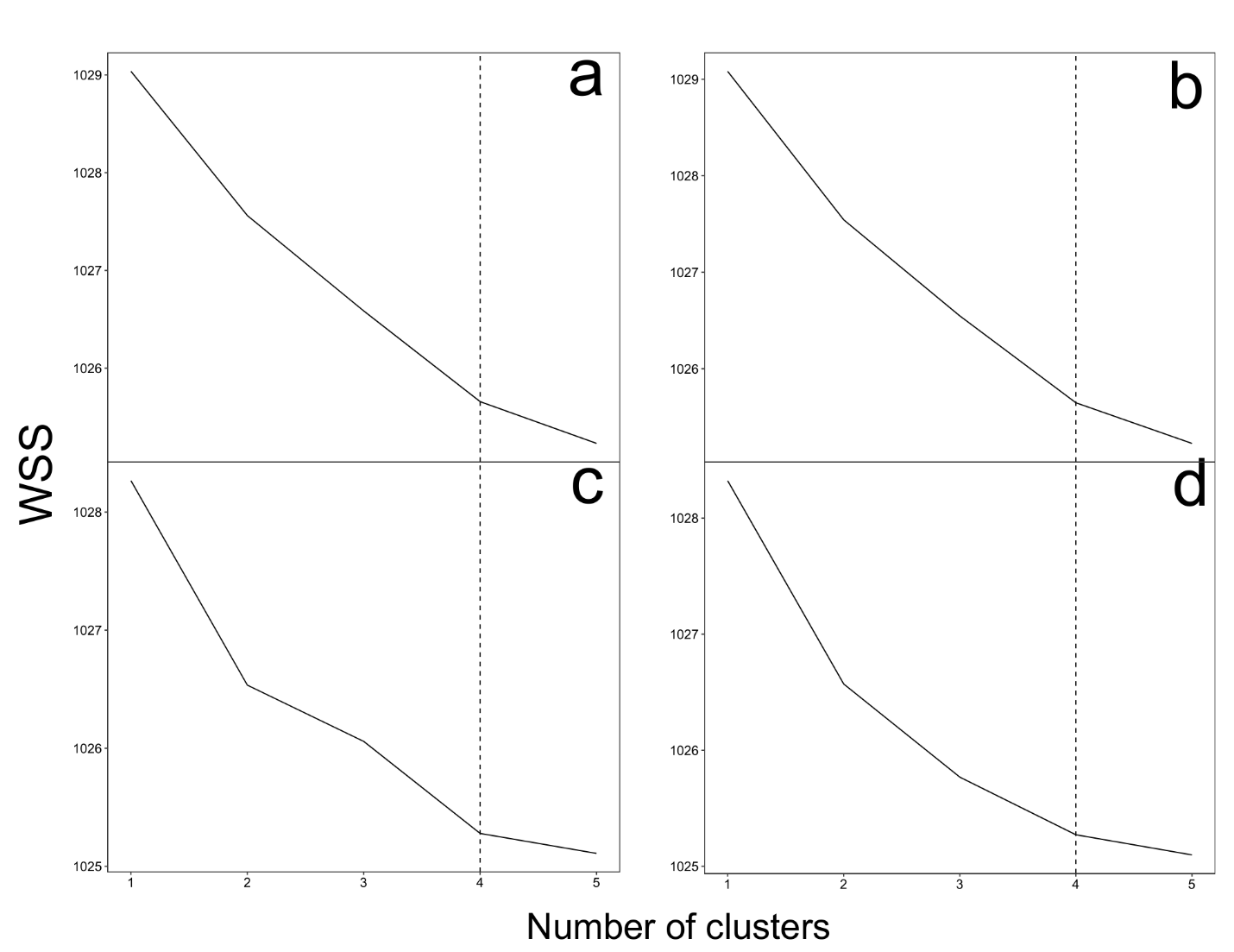


**Supplementary Figure S10.** The optimal number of clusters for the k-means clustering of the significant functional annotation enrichments within six groups attributed to different hosts (human, insect, plant) both all accessory genes and Scoary-reported specificity factors defined by the elbow method. Vertical dotted lines represent the selected number of clusters. The clustering is based on four dissimilarity matrices using the Jaccard coefficient on sets of terms all annotation systems (COG (Cluster of Orthologous Genes) (a), KEGG (Kyoto Encyclopedia of Genes and Genomes), and GO (Gene Ontology)) (**a**) or GO only (**b**) and Szymkiewicz–Simpson metrics on the corresponding data (**c**) (**d**).


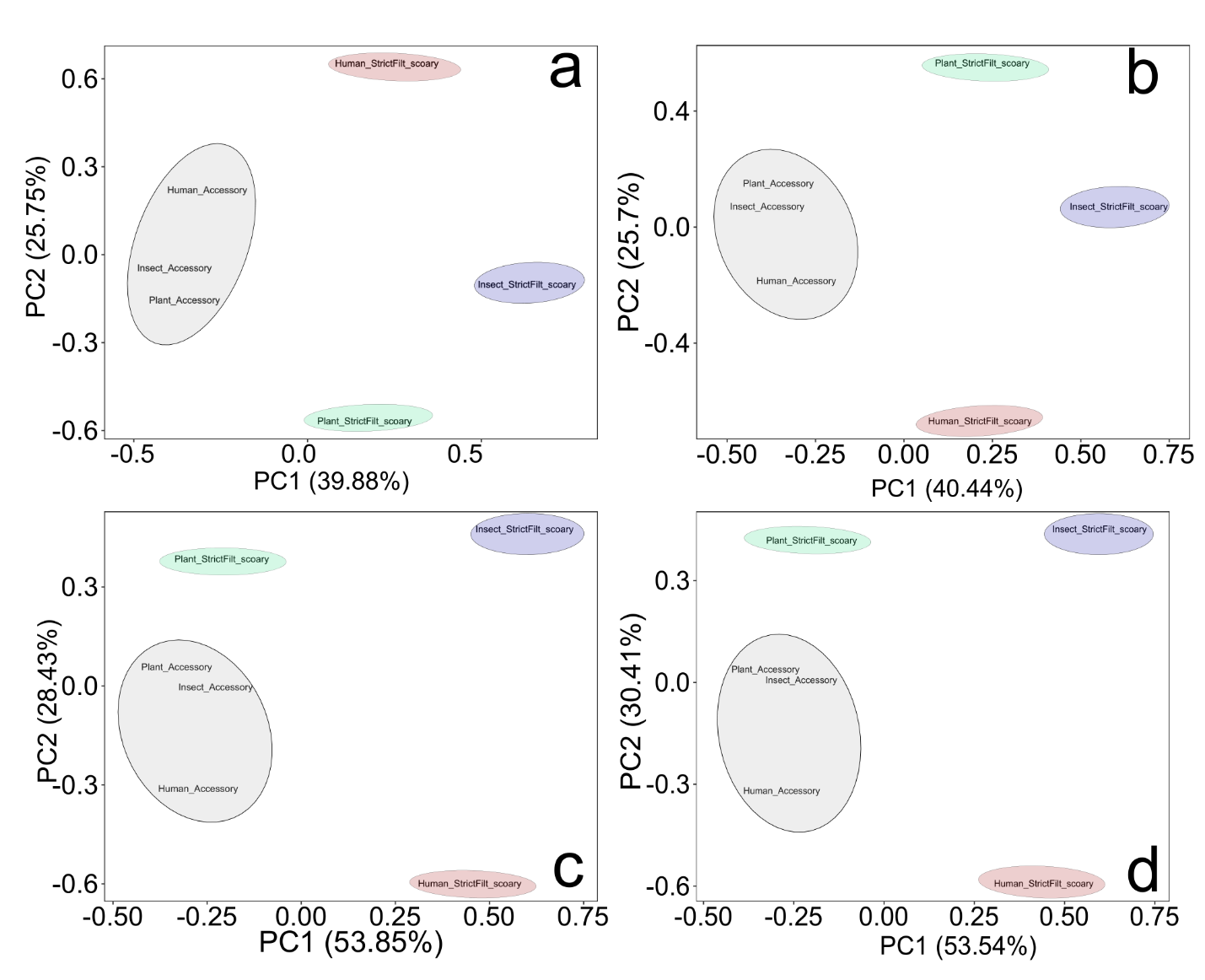


**Supplementary Figure S11.** The k-means algorithm clustering results based on significant functional annotation enrichments within six groups attributed to different hosts (human, insect, plant) both all accessory genes and Scoary-reported specificity factors defined by the elbow method. The clustering is based on four dissimilarity matrices using the Jaccard coefficient on all annotation systems (COG (Cluster of Orthologous Genes) (a), KEGG (Kyoto Encyclopedia of Genes and Genomes), and GO (Gene Ontology)) (**a**) or GO only (**b**) and Szymkiewicz–Simpson metrics on the corresponding data (**c**) (**d**). Grey cluster unifies all groups of accessory genes, while red, blue, and green clusters represent specificity factors related to infecting human, insect, and plant hosts.
